# Multi-omics profiling reveals potential alterations in rheumatoid arthritis with different disease activity levels

**DOI:** 10.1186/s13075-023-03049-z

**Published:** 2023-05-03

**Authors:** Jianghua Chen, Shilin Li, Jing Zhu, Wei Su, Congcong Jian, Jie Zhang, Jianhong Wu, Tingting Wang, Weihua Zhang, Fanwei Zeng, Shengjia Chang, Lihua Jia, Jiang Su, Yi Zhao, Jing Wang, Fanxin Zeng

**Affiliations:** 1 Institute of Basic Medicine and Forensic Medicine, North Sichuan Medical College, Nanchong, Sichuan China; 2 Department of Clinical Research Center, Dazhou Central Hospital, Dazhou, Sichuan China; 3 Department of Rheumatology and Immunology, Sichuan Provincial People’s Hospital, University of Electronic Science and Technology of China, Chengdu, Sichuan China; 4 Department of Rheumatology and Immunology, West China Hospital, Sichuan University, Chengdu, Sichuan China; 5 School of Basic Medical Sciences, Chengdu University of Traditional Chinese Medicine, Chengdu, Sichuan China; 6 Department of Rheumatology and Immunology, Dazhou Central Hospital, Dazhou, Sichuan China; 7 Sichuan Province Orthopaedic Hospital, Chengdu, Sichuan China; 8 Shantou University Medical College, Shantou University, Guangdong, China; 9 Clinical Institute of Inflammation and Immunology (CIII), Frontiers Science Center for Disease-Related Molecular Network, West China Hospital, Sichuan University, Chengdu, Sichuan China; 10 The National Clinical Research Center for Mental Disorders, National Center for Mental Disorders, Beijing Anding Hospital, Capital Medical University, Beijing, China; 11 Department of Big Data and Biomedical AI, College of Future Technology, Peking University, Beijing, 100871 China

**Keywords:** Rheumatoid arthritis, DAS28-ESR, Multi-omics, Lipid metabolism, Whole exome sequencing, Random forest

## Abstract

**Background:**

Rheumatoid arthritis (RA) is a chronic, systemic autoimmune inflammatory disease, the pathogenesis of which is not clear. Clinical remission, or decreased disease activity, is the aim of treatment for RA. However, our understanding of disease activity is inadequate, and clinical remission rates for RA are generally poor. In this study, we used multi-omics profiling to study potential alterations in rheumatoid arthritis with different disease activity levels.

**Methods:**

Fecal and plasma samples from 131 rheumatoid arthritis (RA) patients and 50 healthy subjects were collected for 16S rRNA sequencing, internally transcribed spacer (ITS) sequencing, and liquid chromatography-tandem mass spectrometry (LC–MS/MS). The PBMCS were also collected for RNA sequencing and whole exome sequencing (WES). The disease groups, based on 28 joints and ESR (DAS28), were divided into DAS28L, DAS28M, and DAS28H groups. Three random forest models were constructed and verified with an external validation cohort of 93 subjects.

**Results:**

Our findings revealed significant alterations in plasma metabolites and gut microbiota in RA patients with different disease activities. Moreover, plasma metabolites, especially lipid metabolites, demonstrated a significant correlation with the DAS28 score and also associations with gut bacteria and fungi. KEGG pathway enrichment analysis of plasma metabolites and RNA sequencing data demonstrated alterations in the lipid metabolic pathway in RA progression. Whole exome sequencing (WES) results have shown that non-synonymous single nucleotide variants (nsSNV) of the HLA-DRB1 and HLA-DRB5 gene locus were associated with the disease activity of RA. Furthermore, we developed a disease classifier based on plasma metabolites and gut microbiota that effectively discriminated RA patients with different disease activity in both the discovery cohort and the external validation cohort.

**Conclusion:**

Overall, our multi-omics analysis confirmed that RA patients with different disease activity were altered in plasma metabolites, gut microbiota composition, transcript levels, and DNA. Our study identified the relationship between gut microbiota and plasma metabolites and RA disease activity, which may provide a novel therapeutic direction for improving the clinical remission rate of RA.

**Supplementary Information:**

The online version contains supplementary material available at 10.1186/s13075-023-03049-z.

## Introduction

Rheumatoid arthritis (RA), a complex systemic autoimmune inflammatory disease, could cause severe symptoms like bone destruction and joint deformity [1–3]. However, its pathogenesis remains largely unknown. Meanwhile, RA is highly heterogeneous; RA patients at different stages show different clinical symptoms and respond differently to antirheumatic drugs [4, 5]. Consequently, the disease assessment and the therapy process monitoring of RA are particularly important. Clinically, the disease activity score 28 (DAS28) is one of the most used measurement methods for the disease assessment of RA patients [6, 7]. However, due to the limited understanding of RA disease activity, the rate of RA remission is generally low at present [8]. Thus, there is a continuous need for further study to determine the potential changes in the progression or remission of RA disease activity and then develop a more effective disease assessment model to assist the clinical evaluation of RA patients and improve the clinical remission rate of RA.

At present, omics emerged as a robust research tool in the field of RA, such as metabolomics, microbiomics, and genomics [9–14]. Metabolomics could be used for quantitative analysis of metabolites in organisms which closely related to the occurrence and development of diseases. Previous studies have shown that metabolites were associated with the progression of RA disease and could be applied to distinguish the disease state of RA patients [15]. Microbiomics is a discipline that studies bacteria, lower or higher eukaryotes, and human diseases. The number of microorganisms in the human body is far more than the number of cells, which play an important role in physiological and pathological activities. Previous studies demonstrated that the intestinal flora imbalance could significantly affect the host immune system and cause various diseases, like RA [11, 12, 16]. Furthermore, bacterial pathogens in the gastrointestinal tract could also aggravate disease activity [17]. The whole exome sequencing (WES) can identify rare gene mutations and discover the underlying pathological mechanism [18]. At present, the WES has been applied to study the pathogenesis, disease progression, and genetic variation of RA and found that multiple gene variations are associated with altered immune pathways and increased risk of RA [13, 14]. Currently, the application of multi-omics combined analysis has achieved remarkable success in the diagnosis, treatment, and prognosis of diseases; thus, multi-omics combined analysis hold great potential in RA study. But there is little data about RA acquired by the multi-omics combined analysis.

In this study, we measured the changes in plasma metabolites, gut bacteria, and fungi in RA patients with different disease activity levels. We clarified the relationship between plasma metabolites, gut microbiota, and RA disease activity. Then, our study explored the potential mechanisms of changes in disease activity by multi-omics and provided a promising therapeutic direction for improving the clinical remission rate of RA.

## Methods

### Subject recruitment and sample collection

We recruited 50 healthy control (HC) volunteers and 131 RA patients hospitalized in Dazhou Central Hospital. All RA patients met the diagnostic criteria for American College of Rheumatology/European League Against Rheumatism 2010 and were older than 18 years [1]. Osteoarthritis, psoriatic arthritis, and other autoimmune diseases were excluded. The patients were assessed by professional RA specialists using DAS28-ESR score before admission. Finally, RA patients were further divided into three groups according to DAS28 score: DAS28L (DAS28 ≤ 3.2, *n* = 10), DAS28M (3.2 < DAS28 ≤ 5.1, *n* = 45), and DAS28H (DAS28 > 5.1, *n* = 76).

Clinical information was collected for all subjects, including joint tenderness number, swelling number, c-reactive protein (CRP, mg/L), rheumatoid factor (RF, IU/mL), erythrocyte sedimentation rate (ESR, mm/h), medication use status, and comorbidities, as detailed in Supplementary Table 1. Fasting peripheral venous blood was collected from all subjects in the morning of the second day after admission into the EDTA anticoagulant tube, and fresh stool samples from each subject were collected. Blood and fecal samples were immediately transported to the laboratory, pre-treated according to the standard processing procedures required for sample analysis, and immediately cold stored at − 80 °C. Furthermore, we included an external validation cohort of 93 people, who met the same inclusion and exclusion criteria as the discovery cohort and were also divided into healthy controls (HC) group (*n* = 20), DAS28L group (*n* = 21), DAS28M group (*n* = 23), and DAS28H group (*N* = 29). Similarly, we collected their clinical information as detailed in Supplementary Table 2.

### Fecal sample total DNA extraction and sequencing

Total DNA was extracted from the stool samples of the subjects by a biotechnology company (Magi Bio, China), and 16S of bacteria were amplified from the extracted DNA with primers 338F ACTCCTACGGGAGGCAGCAG and 806R GGACTACHVGGGTWTCTAAT rRNA gene fragment (V3-V4); the ITS of fungi was amplified from the extracted DNA with primers ITS1F CTTGGTCATTTAGAGGAAGTAA and ITS2R GCTGCGTTCTTCTTCTTCATCGATGC. Finally, the obtained amplicon sequence variation (ASVs) was extracted and leveled according to the minimum sample sequence number. Detailed analysis method is shown in Supplementary material 1.

### Non-targeted metabolomics

The subjects’ plasma samples were analyzed for non-targeted metabolomics by UPLC-MS/MS by a metabolic company (MagiBio, China). Metabolites with missing values greater than 80% in each group of the original values and relative standard deviation (RSD) greater than 30% in QC samples were removed. Meanwhile, the missing values in the original values were filled with minimum values to preprocess the expression data of the original detection results. Detailed description of metabolomics analysis method is shown in Supplementary material 2.

### RNA sequencing

The subjects’ PBMC samples were extracted with total RNA using standard methods by a biological company (Norogene, Beijing); RNA integrity and total amounts were assessed with Agilent 2100 BioAnalyzer (Agilent Technologies, CA, USA). Library preparation was done by NEBNext® Ultra™ RNA Library Prep Kit for Illumina®. After the library is qualified, Illumina NovaSeq 6000 was used for sequencing and 150-bp paired-end readings were generated. The reference genome index was constructed by HISAT2 (v2.0.5), and paired-end clean reads were aligned with the reference genome.

### Whole exome sequencing

In brief, DNA was extracted from the peripheral blood mononuclear cells (PBMC) of subjects according to QIAamp DNA Blood Mini Kit (Qiagen, Germany) instructions. Library preparation and purification were done according to the Illumina® DNA Prep with Enrichment (Tagmentation), Illumina Exome Panel, and Agencourt AMPure XP (Beckman, USA) instructions, respectively. Sequencing was performed using NextSeq 2000. After the raw data were obtained by sequencing, the information analysis and screening process was carried out based on the HG19 human reference genome. The analysis process mainly includes (1) sequencing data quality assessment, (2) mutation detection to find SNP and InDel, and (3) mutation site screening.

### Statistical analysis

Partial analysis results of non-target metabolite analysis, bacterial diversity, and fungal diversity were analyzed via online platform of Majorbio cloud platform (www.majorbio.com), including orthogonal partial least-squares discriminant analysis (OPLS-DA), Kyoto Encyclopedia of Genes and Genomes (KEGG) pathway enrichment, α diversity analysis, linear discriminant analysis (LDA) effect size (LEfSe) analysis, and Phylogenetic Investigation of Communities by Reconstruction of Unobserved States (PICRUSt2) function prediction analysis. Analysis of all results was performed by using GraphPad Prism (v8.0), SPSS Statistics (V.25.0.0.0), STRING (https://cn.string-db.org), Cytoscape (V3.9.1), and R software (V3.6.2).

## Results

### Analysis workflow

**Fig. 1:**
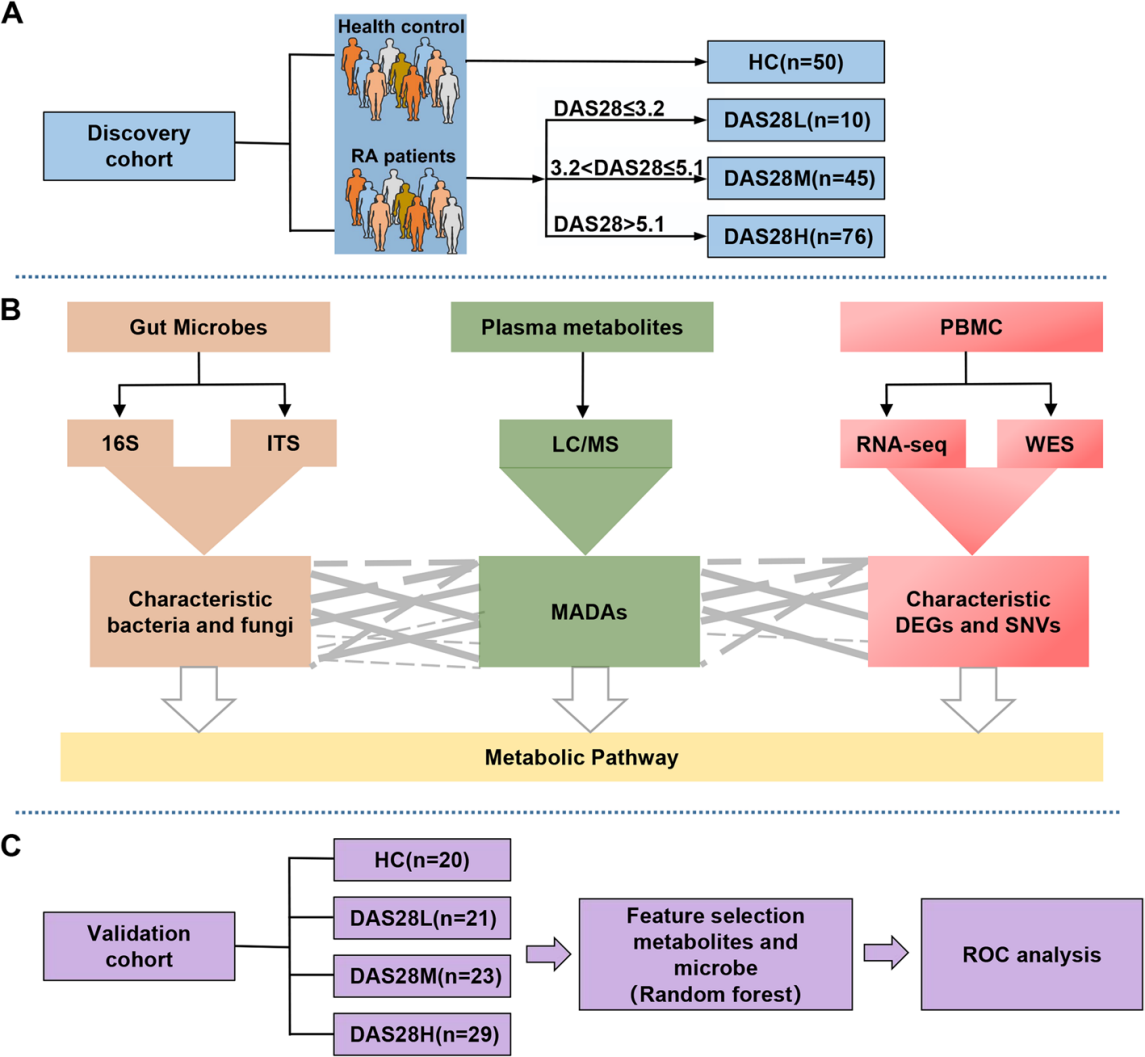
Flow chart of experiment. **A** Population distribution for subjects in the discovery cohort. **B** Multi-omics analysis of RA with different disease activity levels. **C** ROC analysis of the external validation cohort in a classification model constructed through a random forest. RA, rheumatoid arthritis; HC, healthy controls; DAS28, Disease Activity Score 28; ITS, Internally Transcribed Spacer; LC–MS, liquid chromatograph mass spectrometer; MADAs, metabolites associated with disease activity; RA-seq, RA sequencing; WES, whole exome sequencing; DEGs, differentially expressed genes; nsSNV, non-synonymous single nucleotide variation; ROC, receiver operating characteristic curves

The entire analytical process is summarized in Fig. 1 to explore the association between plasma metabolites, gut microbes, transcript levels, non-synonymous single nucleotide variants (nsSNV), and RA disease activity. The contents include the discovery cohort population distribution (Fig. 1A) and multi-omics analysis of RA with different disease activity levels (Fig. 1B). The random forest model performs receiver operator characteristic curve (ROC) analysis in the external validation cohort (Fig. 1C).

### Changes of characteristic plasma metabolites in RA with different disease activity levels

To identify the differential metabolic characteristics among the four groups, we performed a non-targeted metabolomics test on fasting plasma of all participants. A total of 281 metabolites (cationic modes: 133, anionic modes: 148) were annotated in HMDB and Metlin databases. The selection of significantly different metabolites was determined based on the variable importance for the projection (VIP) value of OPLS-DA model and *P* value by Wilcoxon test; VIP > 1, *p* < 0.05 were defined as significantly different metabolites. Compared with the HC group, there were 28, 38, and 49 different metabolites in the three disease groups (Supplementary Fig. 1A-C). Comparison of DAS28M vs. DAS28L, and DAS28H vs. DAS28M with 8 and 9 metabolites is shown in Supplementary Fig. 1D, E. The Venn diagram showed the same and specific differential metabolites between groups, suggesting that as the disease activity increases, the differential metabolites also increase (Supplementary Fig. 1F, G).

**Fig. 2:**
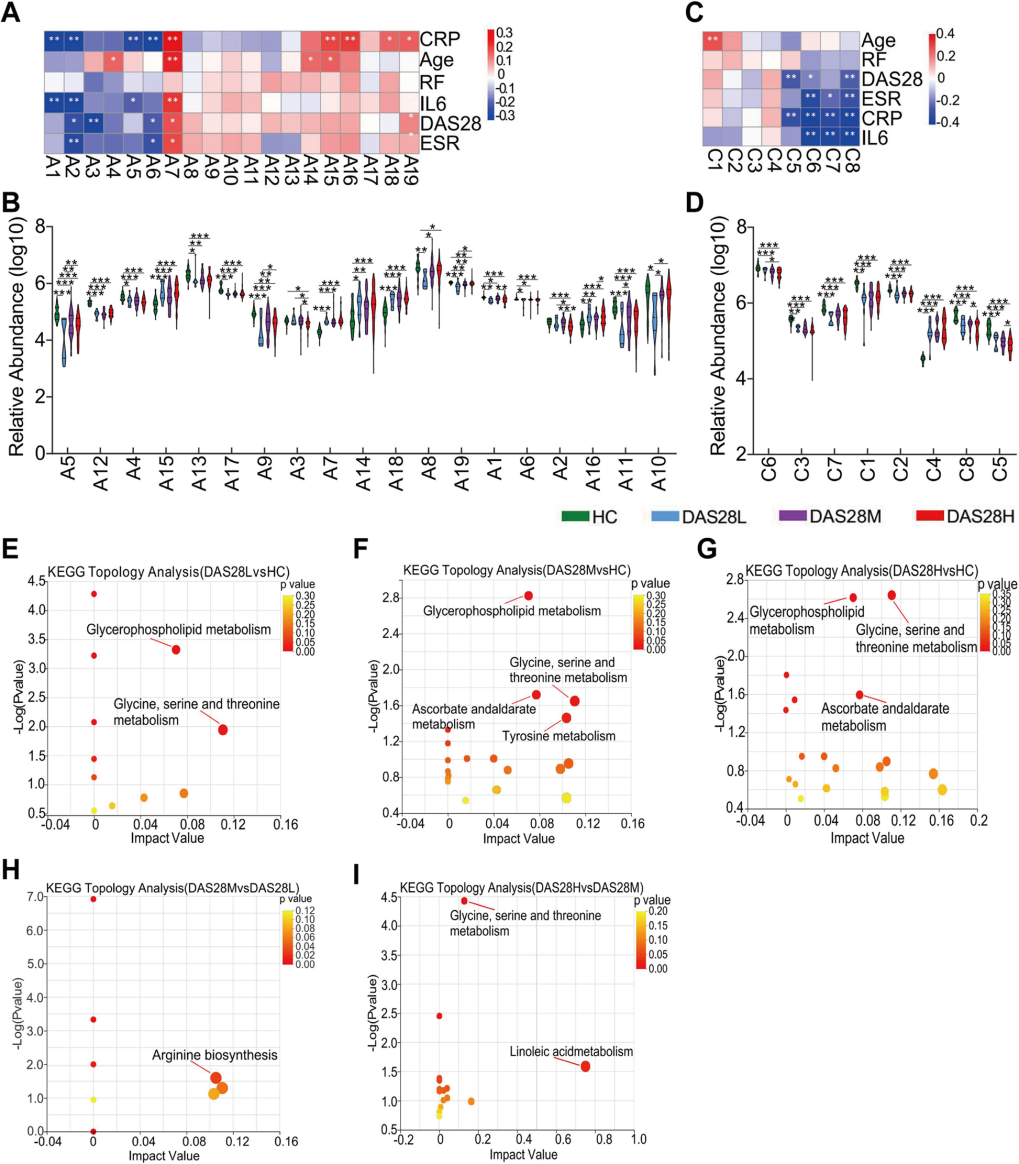
Analysis of characteristic plasma metabolites in RA with different disease activity levels. **A**, **C** Heat maps of correlation between characteristic metabolites in anionic (**A**) and cationic (**C**) modes and clinical indicators of RA. Spearman was used for correlation analysis. Red represents a positive correlation and blue represents a negative correlation. **B**, **D** The box plot showed that characteristic metabolites of anionic (**B**) and cationic (**D**) modules significantly changed between RA with different disease activity levels according to Wilcoxon rank-sum test. Boxes represent the inter-quartile ranges, and lines inside the boxes denote medians. **E**–**I** Differential metabolites of DAS28L vs. HC (**E**), DAS28M vs. HC (**F**), DAS28H vs. HC (**G**), DAS28M vs. DAS28L (**F**), and DAS28H vs. DAS28M (**I**) were enriched in KEGG pathway. The color bars indicate enrichment significance levels. *P*-value, *p < 0.05, **p < 0.01, ***p < 0.001

Next, based on Spearman correlation analysis, heat maps showed the relationship between clinical parameters and characteristic difference metabolites (Supplementary Table 3). L-threonine, linoleic acid, deoxycholic acid, docosahexaenoic acid, 1,4-dihydroxybenzene, and L-tryptophan (A1-A6) have a negative relationship with inflammatory biomarkers (CRP, ESR, and IL-6) and DAS28 scores in anion mode (Fig. 2A). Their relative expression abundance in three disease groups decreased (Fig. 2B). However, the reverse occurred for D-galacturonic acid (A7) (Fig. 2A, B). MG (18:2(9Z,12Z)/0:0/0:0), 1-oleoyl-sn-glycero-3-phosphocholine (OGPC), 1-stearoyl-2-hydroxy-sn-glycero-3-phosphocholine (18:0LYSO-PE), and glycerophosphocholine (GPC) (C5-C8) also have a negative relationship with inflammatory biomarkers and DAS28 score (Fig. 2C). Their relative expression abundance in three disease groups decreased (Fig. 2D). We performed KEGG pathway enrichment analysis with differential metabolites to explore the changes in metabolic pathways in RA progression. The metabolites in the disease group were mainly enriched in glycerophospholipid metabolism and glycine, serine, and threonine metabolism pathways compared with HC groups (Fig. 2E–G). Meanwhile, differential metabolites between the disease groups were enriched in arginine biosynthesis and linoleic acid metabolism pathways (Fig. 2H, I). These results suggest that metabolic pathways change during RA progression, especially glycerophospholipid and linoleic acid pathways, which were defined as lipid metabolic pathways.

### Changes in gut microbiota composition among RA with different disease activity levels

**Fig. 3:**
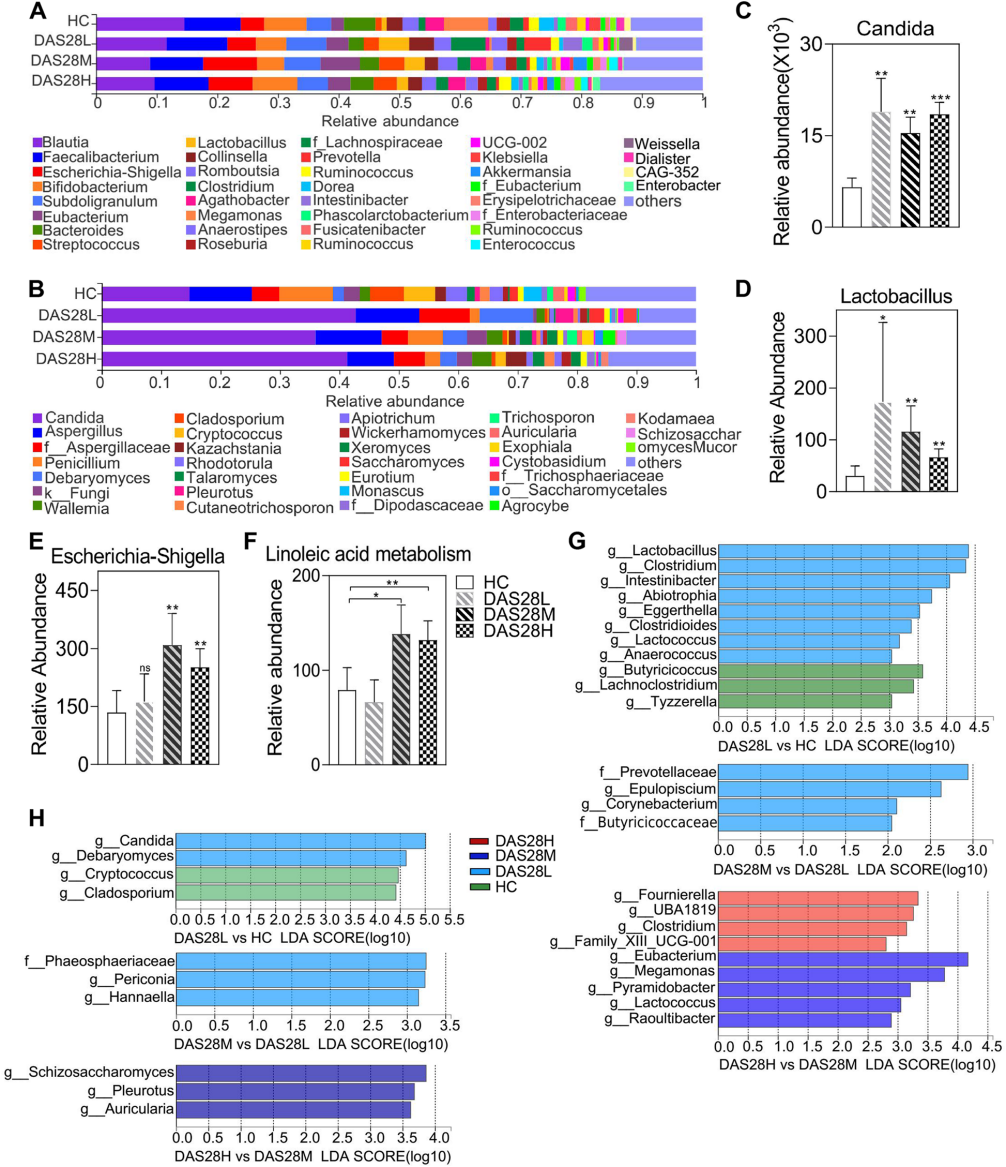
Changes of gut bacteria and fungi between RA with different disease activity levels. **A**, **B** The distribution plot of relative abundance at the genus level of bacteria (**A**) and fungi (**B**). **C**–**E** Relative abundance of *Candida* (**C**), *Lactobacillus* (**D**), and *Escherichia*-*Shigella* (**E**) in the HC group and RA with different disease activity levels groups. **F** Intestinal bacterial PICRUSt2 function predicts changes in linoleic acid metabolism pathway in RA progression. **G**, **H** LefSe analysis was used to identify highly differentiated taxa for RA gut bacteria (**G**) and fungi (**H**) with different disease activity levels and to display LDA scores. The bar chart shows the mean value of each group. Error bars represent the standard error of the mean values. RA, rheumatoid arthritis; HC, healthy controls; PICRUSt2, Phylogenetic Investigation of Communities by Reconstruction of Unobserved States; LefSe, linear discriminant analysis (LDA) effect size; *P*-value, *p < 0.05, **p < 0.01, ***p < 0.001. NS, not significant

Intestinal microbiome composition of all subjects was detected by 16S rRNA and ITS sequencing; we found that, at the phylum level of gut bacteria, with the increase of disease activity, the relative abundance proportion of *Firmicutes* decreased gradually, while *Proteobacteria* was the opposite (Supplementary Fig. 2A). Gut fungi were mainly composed of *Ascomycota* and *Basidiomycota*. *Ascomycota* was enriched in the disease groups, while *Basidiomycota* was enriched in the HC groups (Supplementary Fig. 2B). We visualized the species composition of bacteria and fungi at the genus level and found that other genera also showed various degrees of change (Fig. 3A, B). *Candida* in fungi increased significantly in the disease group, but there was no significant difference among the disease groups (Fig. 3C). *Penicillium* was reduced in the disease group, especially in the das28H group (Supplementary Fig. 2C). *Lactobacillus* is considered an intestinal probiotic, and its relative abundance in the disease group was significantly higher than that in the HC group, but gradually decreased among the disease groups (Fig. 3D). *Escherichia-Shigella* was significantly higher than HC groups in the disease group, and there was a significant difference between DAS28M, DAS28H groups, and HC groups (Fig. 3E).

We also studied changes of gut microbial diversity. By the Venn analysis of ASV in the four groups, intestinal bacterial species diversity decreased in the DAS28L group and then gradually increased. Meanwhile, we also found that the number of specific bacteria increased with the increase of disease activity (Supplementary Fig. 2D). We also found the same results in gut fungi (Supplementary Fig. 2E). In our results, only the alpha diversity indices of intestinal fungi Shannon even, Simpson, and Simpson even showed significant differences between the disease group and HC, while there was no difference among the disease group (Supplementary Fig. 2F-H). Then, we performed PICRUSt2 function prediction on bacterial 16S ribosomal ribonucleic acid amplicon sequencing data, and the results showed that linoleic acid metabolic pathway was significantly enhanced in DAS28M and DAS28H groups (Fig. 3F). However, glycerophospholipid metabolic pathway and arachidonic acid (AA) metabolic pathway were changed in the disease group, but there was no significant difference (Supplementary Fig. 2I, J).

To further study the potential differences between the HC group and RA with different disease activity levels, the LEfSe method was used to analyze the composition of intestinal microbes. At the bacterial genus level, *Lactobacillus*, an unidentified *Prevotella*, and *Eubacterium* were the bacteria with the highest LDA scores in DAS28L vs. HC DAS28M vs. DAS28L and DAS28H vs. DAS28M groups, respectively (Fig. 3G). Similarly, the fungi with the highest LDA scores were *Candida*, an unidentified *Phaeosphaeriaceae*, and *Schizosaccharomyces*, respectively (Fig. 3H).

### Transcriptome analysis of RA suggests changes in lipid metabolism pathways

**Fig. 4:**
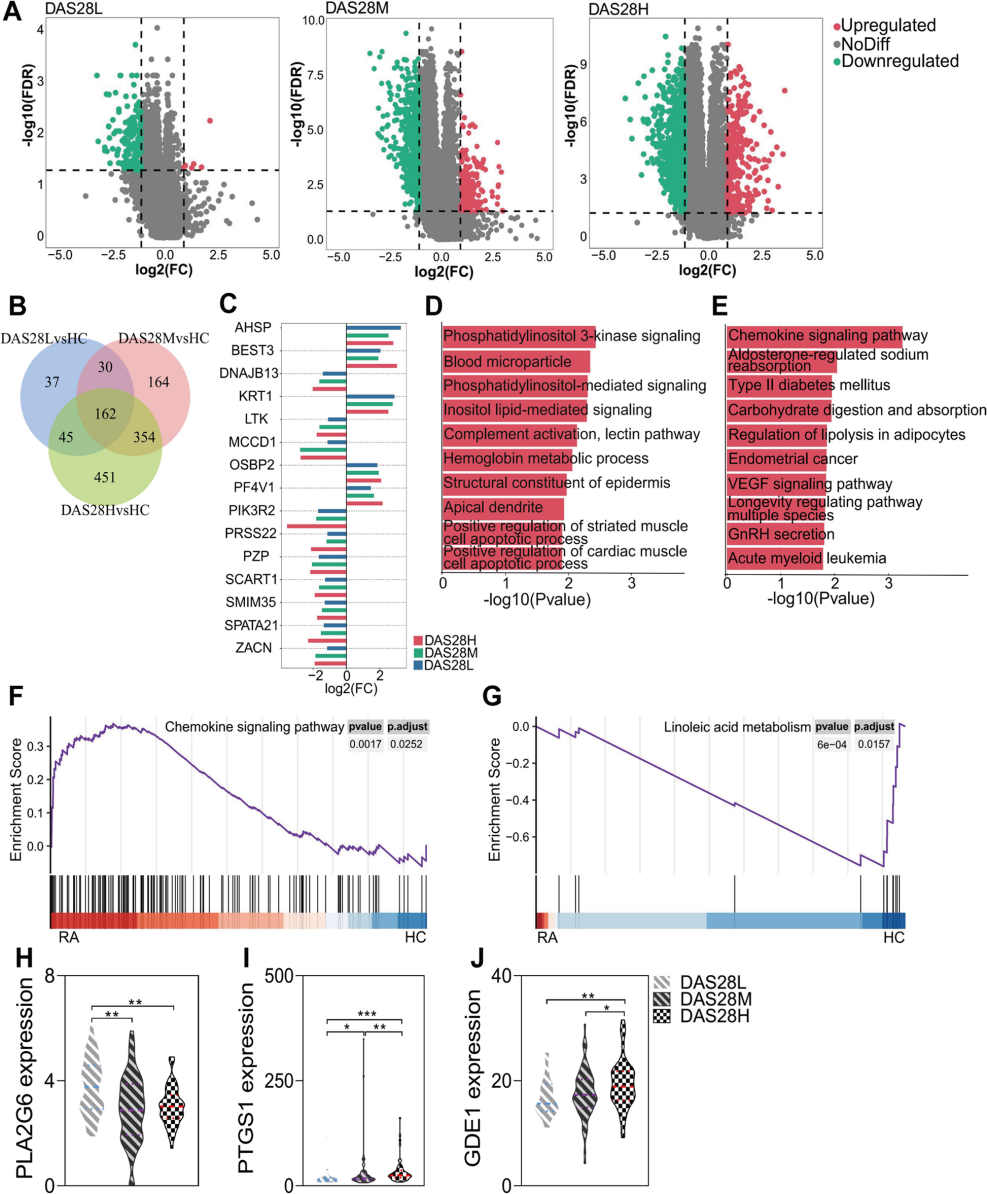
Transcriptome analysis of RA suggests changes in lipid metabolism pathways. **A** Volcano plots showing DEGs in RA with different disease activity, respectively. Red indicated upregulated genes, and green indicated downregulated genes. **B** Venn diagram showing 162 DEGs in common across RA with different disease activity. **C** Fold change of DAS28-related genes in RA with different disease activity. **D**, **E** Bar graphs showing the top 10 most significant pathways of DAS28-related genes in Gene ontology term (**D**) and Kyoto Encyclopedia of Genes and Genomes (**E**) enrichment analysis. **F**, **G** GSEA analysis showing DEGs of RA vs. HC were upregulated in the chemokine signaling pathway (**F**) and downregulated in the linoleic acid metabolism pathway (**G**). **H**, **J** Expression of PLA2G6 (**H**), PTGS1 (**I**), and GDE1 (**J**) gene in lipid metabolism pathway in RA progression. The bar chart shows the mean value of each group. Error bars represent standard error of the mean values. RA, rheumatoid arthritis; HC, healthy controls; DEGs, differentially expressed genes; *P*-value, *p < 0.05, **p < 0.01, ***p < 0.001

We sequenced RNA-seq data from a large number of RA patients with different disease activities and used principal component analysis to demonstrate that these disease groups were similar (Supplementary Fig. 3A). Compared with the HC group, the differentially expressed genes (DEGs) in different disease groups increased with the increase in disease activity (Fig. 4A). Meanwhile, they had 162 shared DEGs, including 11 upregulated genes and 151 downregulated genes (Fig. 4B). A total of 15 genes were associated with DAS28 score (Spearman, |*R*|> 0.1, *P* < 0.05), and their fold change was altered in different disease groups (Fig. 4C, Supplementary Table 4). Genes related to disease activity were mainly enriched in biological processes related to phosphatidylinositol 3-kinase signaling (Fig. 4D). The most affected pathway was the chemokine signaling pathway (Fig. 4E). Similarly, genes related to chemokine signaling pathway were significantly upregulated in RA by GSEA analysis (Fig. 4F). In addition, we also found that linoleic acid metabolism genes were significantly downregulated in RA (Fig. 4G). The same results were also observed for glycine, serine, and threonine metabolism; glycerophospholipid metabolism; and arachidonic acid metabolism as linoleic acid metabolism (Supplementary Fig S3B-D).

Then, to demonstrate the changes of lipid metabolism pathways in the progression of RA, we further analyzed 127 genes of three lipid metabolism pathways and found 61 differential lipid metabolism-related genes (LMRGs) (FDR < 0.05, Supplementary Fig. 3E). Among them, 9 differential LMRGs were associated with differential metabolites of lipid metabolism pathway (3 up-regulated and 6 down-regulated). Compared with the DAS28L group, PLA2G6Z was significantly decreased in DAS28M and DAS28H groups (Fig. 4H). However, PTGS1 increased gradually with the increase in disease activity (Fig. 4I). Another LMRG, GDE1, was significantly increased only in the DAS28H group (Fig. 4J). Overall, transcriptional data analysis indicated that lipid metabolic pathways were altered during RA progression.

### nsSNV of the HLA-DRB1 and HLA-DRB5 gene locus were associated with the disease activity of RA

**Fig. 5:**
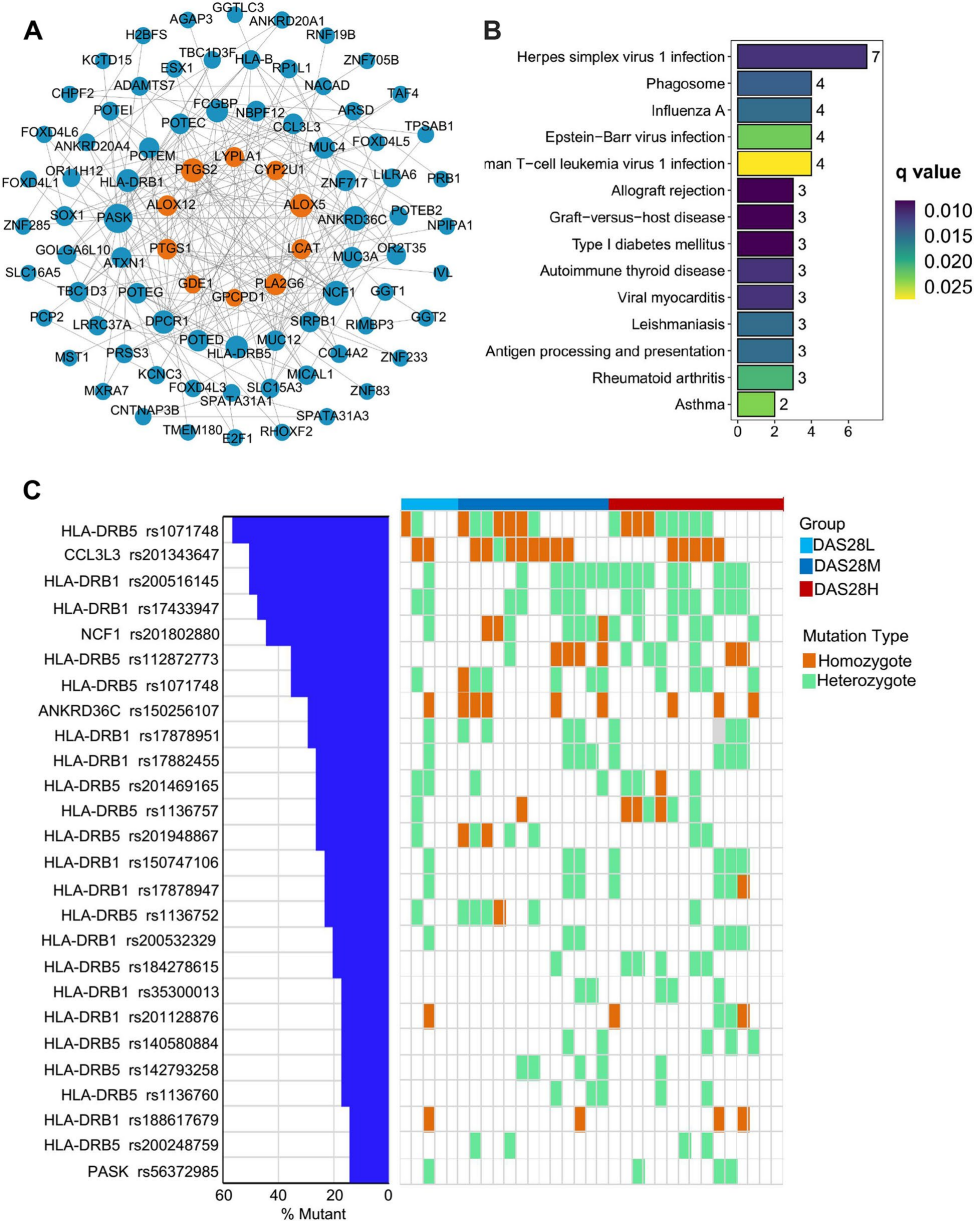
nsSNV of the HLA-DRB1 and HLA-DRB5 gene locus were associated with the disease activity of RA. **A** PPI analysis of genes with nsSNV mutation rate greater than 10% and genes involved in lipid metabolism pathways. The blue dots represent genes with RA PMBC nsSNV mutation rate greater than 10%, and the yellow dots represent genes associated with lipid metabolism pathways. The size of the point represents the node size of the gene in PPI. **B** KEGG pathway enrichment analysis bars of related genes in PPI analysis. Color bars indicate enrichment significance. **C** Mutation rates of nsSNV in related genes in different disease activity groups. nsSNV, non-synonymous single nucleotide variation; RA, rheumatoid arthritis; PPI, protein–protein interaction; PMBC, peripheral mononuclear blood cell

We collected PMBC of 20 healthy subjects and 33 RA patients for WES analysis. A total of 97 nsSNV genes with mutation rate greater than 10% in exons were selected for protein–protein interaction (PPI) analysis on STRING with key protease genes in three lipid metabolism pathways. Finally, a total of 74 genes participated in the protein interaction network of lipid metabolism (Fig. 5A). KEGG and GO enrichment analysis of these 74 genes showed that 3 genes were enriched in RA, namely HLA-DRB1, HLA-DRB5, and CCL3L3 (Fig. 5B). Meanwhile, leukotriene D4 metabolic process and leukotriene biosynthetic process are also significantly enriched (Supplementary Fig. 4A). Per-Arnt-Sim Kinase has the largest number of connections in the network, followed by neutrophil solute factor 1 (NCF1) and ANKRD36C. NCF1 was the gene with the largest number of nodes interacting with lipid metabolism genes (Supplementary Fig. 4B). According to the statistical analysis of mutation rates of the six genes mentioned above in disease activity degree, the mutation rate of HLA-DRB5 rs1071748 was the highest, and the mutation rates of the three groups were 40.0% (DAS28L), 53.8% (DAS28M), and 60.0% (DAS28H) respectively (Fig. 5C). Meanwhile, some gene loci of HLA-DRB1 and HLA-DRB5 were only mutated in the DAS28M groups and DAS28H groups. Similarly, we also found that the mutation rate of NCF1 rs201802880 in the DAS28L groups (20.0%) was lower than in the DAS28M groups (53.8%) and DAS28H groups (40.0%). Therefore, we hypothesized that nsSNV of the HLA-DRB1 and HLA-DRB5 gene locus may be associated with RA with the disease activity.

### Multi-omics analysis revealed the relationship between gut microbiota, plasma metabolites, LMRGs, and exons nsSNV and RA disease activity

**Fig. 6:**
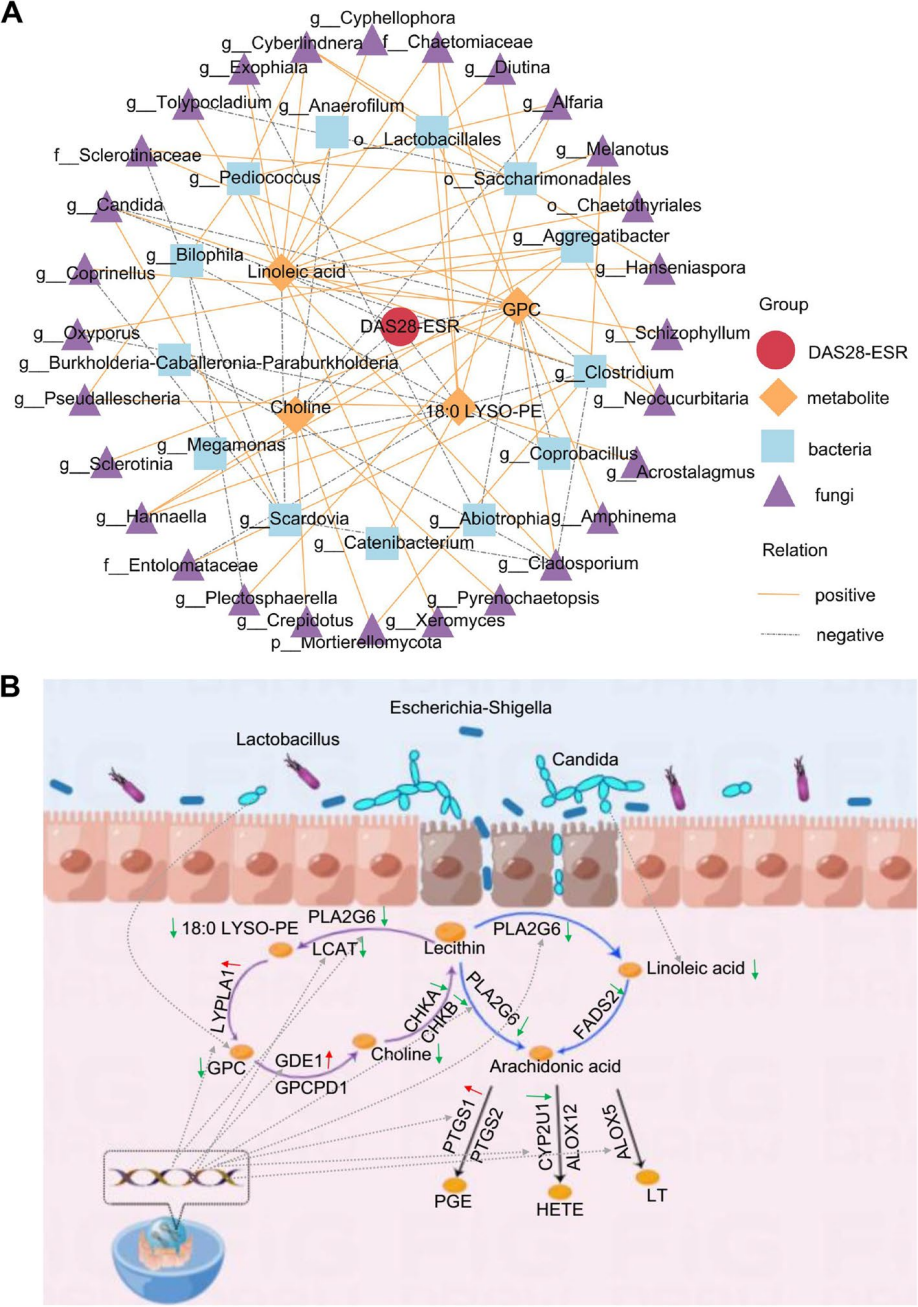
Multi-omics analysis revealed the relationship between gut microbiota, plasma metabolites, LMRGs, and exons nsSNV and RA disease activity. **A** Association analysis of plasma metabolites and gut microbiota with RA disease activity. Red dots represent DAS28-ESR scores, yellow diamonds represent metabolites, cyan squares represent intestinal bacteria, purple triangles represent intestinal fungi, solid yellow lines indicate positive associations, and gray dotted lines indicate negative associations. All lines indicate significant associations (*p* < 0.05). **B** RA pathway change pattern diagram. Plasma metabolites, gut microbe, and nsSNV together constitute a regulatory network for RA disease activity. LMRGs, lipid metabolism-related genes; nsSNV, non-synonymous single nucleotide variation; RA, rheumatoid arthritis; PGE, prostaglandin E; LT, leukotriene; HETE, hydroxyeicosatetraenoic acid

In Fig. 2, metabolites enriched in glycerophospholipid and linoleic acid metabolic pathways were negatively correlated with RA disease activity. To further study the effect of gut microbe on metabolites associated with disease activity (MADAs) in lipid metabolism pathway, the bacteria and fungi with differences between the HC group and disease group and disease activity-related metabolites were used for correlation analysis. There were 28 fungal genera and 13 bacterial genera were significantly correlated with MADAs, and they were also correlated among themselves (Spearman’s correlation analysis, *p* < 0.05, Fig. 6A, Supplementary Fig. 5). We used RA transcription data to demonstrate that genes related to lipid metabolism were altered in RA progression and correlated with disease activity. Meanwhile, the genes of nsSNV participated in the interaction network of key proteins in lipid metabolism. Therefore, we reasoned that plasma metabolites, gut microbe, and nsSNV together constitute a regulatory network for RA disease activity (Fig. 6B).

### Identification and prediction of RA with different disease activities based on plasma metabolites and gut microbiota

**Fig. 7:**
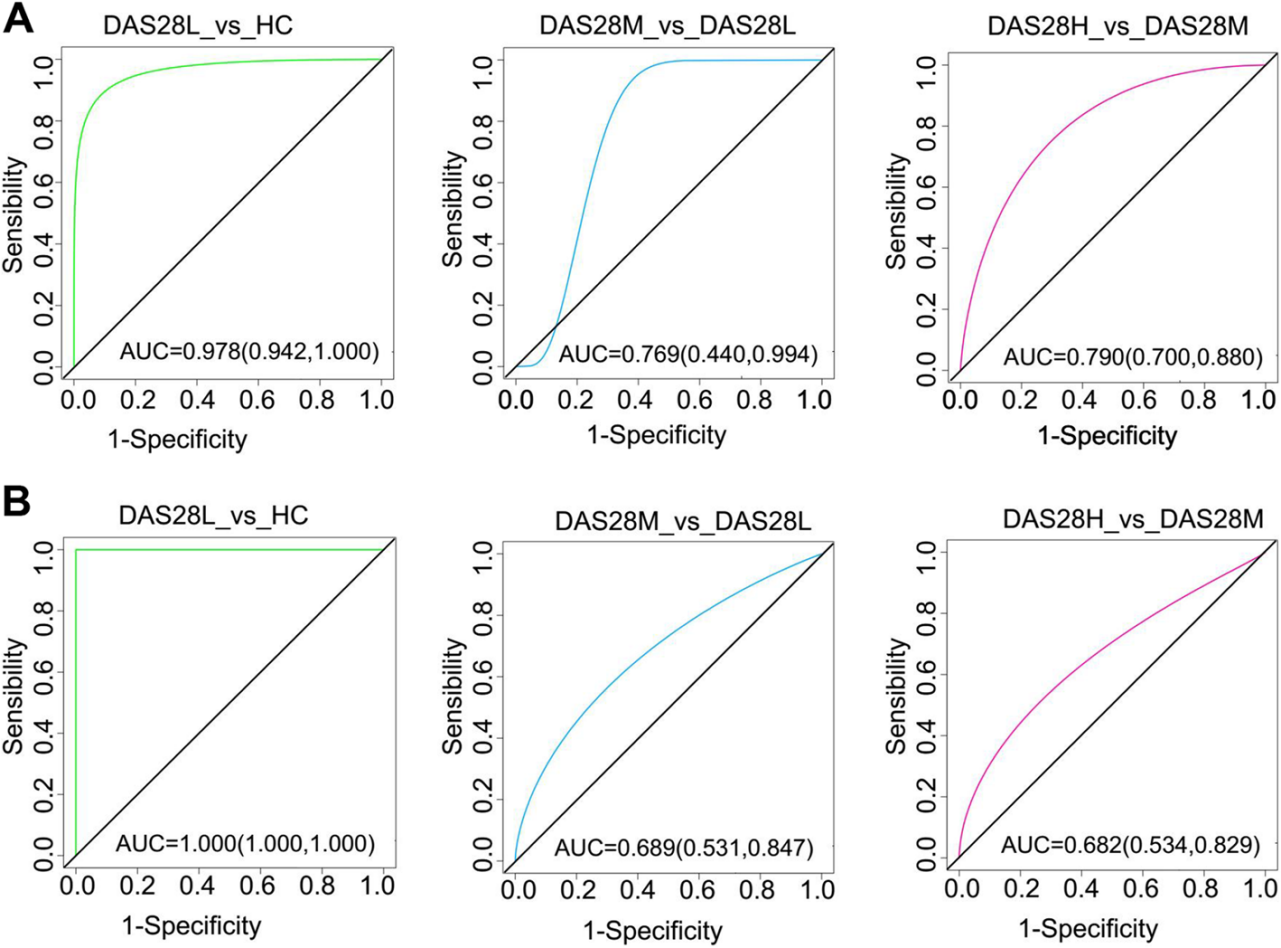
Diagnostic outcomes of RA disease activity are shown by the receiver operating characteristic curve (ROC). **A** ROC of three random forest models (DAS28L vs. HC, DAS28M vs. DAS28L, and DAS28H vs. DAS28M) in the discovery cohort. **B** ROC of three random forest models in the external validation cohort

The random forest algorithm was used to select characteristic parameters for the differential metabolites, bacteria, and fungi of HC groups and three disease groups for classification model construction, and the area under the curve (AUC) value determines the number of important characteristic parameters of the model. In DAS28L vs. HC, DAS28M vs. DAS28L, and DAS28H vs. DAS28M of the three models, the top 5, 4, and 5 characteristic parameters of importance were selected according to AUC values respectively to build the model (Supplementary Fig. 6A-F). ROC analysis was performed on the three models in the discovery cohort, and their AUC values were 0.987 (0.942,1.000), 0.769 (0.440,0.994), and 0.790 (0.700,0.880), respectively (Fig. 7A). Meanwhile, we also included an external validation cohort with the same criteria as the discovery cohort. Finally, the three models were verified by an external validation cohort, and their AUC values were 1.000 (1.000, 1.000), 0.689 (0.531, 0.847), and 0.682 (0.534, 0.829), respectively (Fig. 7B).

## Discussion

In this study, we explored alterations in plasma metabolites and gut microbiota in RA patients with different disease activity levels using 16S rRNA, ITS sequencing, and non-targeted metabolomics analysis. We observed that RA patients with different levels of disease activity exhibited significant changes in both their gut microbiota and plasma metabolites. Plasma metabolites showed relationships with gut bacteria and fungi, as well as a significant link with the DAS28 score. Furthermore, we discovered that RA patients with varying disease activity had varying degrees of transcriptional and DNA-level alterations, and these abnormalities were connected with illness activity. Finally, in both the discovery cohort and the external validation cohort, the disease classifier based on gut microbiota and plasma metabolites was helpful for identifying and predicting RA patients with different disease activities.

In recent years, accumulating evidence indicated that the progression of RA is closely associated with gut microbes [16, 19]. We analyzed fecal bacterial diversity by 16S rRNA sequencing and concluded that the diversity of HC and RA with different disease activity levels did not change significantly, which was consistent with the conclusion by Yu et al. [20]. *Firmicutes* is a natural barrier of the intestinal mucosa, which mainly plays a role in maintaining structural integrity. *Firmicutes* has specific functions in immune regulation and resistance to pathogens, and their reduction can lead to intestinal microbial dysbiosis, which is related to metabolic diseases [21, 22]. In our study, the relative proportion of *Firmicutes* in RA with different disease activity levels gradually decreased, signaling that the reduction of *Firmicutes* leads to further dysbiosis and destruction of the intestinal mucosa, as well as decreased immune regulatory function and increased disease activity. *Escherichia-Shigella* in *Proteobacteria* belongs to gram-negative bacillus. Additionally, lipopolysaccharide, the main component of its cell wall, can further increase intestinal mucosal permeability and aggravate inflammatory response [23]. Jeong et al., based on function prediction analysis of PICRUSt2, showed that genes related to lipopolysaccharide biosynthesis were significantly enriched in RA patients [24]. In this study, *Escherichia-Shigella* was significantly enriched in RA and increased in the DAS28M and DAS28H groups, indicating that intestinal mucosal structure would be further damaged with exacerbation of disease.

Similarly, *Candida*, another important intestinal mucosal microorganism, is also associated with intestinal mucosal structure. In the inflammatory microenvironment of intestinal diseases, the relative abundance and diversity of fungi, especially *Candida*, increased significantly [25]. We found that the relative abundance of *Candida* also increased significantly in RA, which may further induce gut dysbiosis, epithelial cell damage, and invasive infection [26, 27].

The gut probiotics, including *Lactobacillus* and *Bifidobacterium*, that inhabited the human gut could regulate the immune function of the mucosa and maintain the balance of the gut flora [28]. Researchers found that after oral administration of *Lactobacillus* in collagen-induced arthritis (CIA) model rats, the expression of pro-inflammatory factors decreased while the expression of anti-inflammatory factors increased, consequently improving score inflammation [29]. In another study, *Lactobacillus* was used to restore gut flora imbalance and avoid bone destruction in adjuvant-induced arthritis (AIA) model rats [30]. The relative abundance of *Lactobacillus* in RA has been reported to be increased as well as decreased in previous studies [31, 32]. Our results were consistent with the former. Meanwhile, we also found that the decrease of *Lactobacillus* increased the DAS28 score. We speculate that the increase of *Lactobacillus* in early RA could improve the expression level of anti-inflammatory factors, thus dampening down inflammation. However, in the progress of RA, the gut microbiota was further disordered, and the relative abundance of *Lactobacillus* decreased, resulting in decreased expression of anti-inflammatory factors, and promoting RA disease activity.

The glycerophospholipid metabolism, linoleic acid metabolism, and AA metabolism together constitute lipid metabolism pathways. We observed that differential metabolites in the disease and HC groups were all enriched in the glycerophospholipid metabolism pathway, and mainly 18:0 LYSO-PE, GPC, and choline in this pathway decreased during RA progression. 18:0 LySO-PE and GPC were negatively correlated with the DAS28 score, suggesting that decreased function of glycerophospholipid metabolic pathway was associated with disease activity in RA progression. The results of bacterial function prediction also supported this conclusion. OGPC was also negatively correlated with DAS28 score, and its expression decreased with the progression of RA. In our previous studies, OGPC reduction has been identified as a promoting factor of increased IL-6 expression [33]. We were also concerned about the differential metabolites enriched linoleic acid pathway in the DAS28H vs. DAS28M group, and the expression level of linoleic acid decreased during RA progression. Linoleic acid is an unsaturated fatty acid. Currently, there is controversy about the relationship between linoleic acid and inflammation. First, several investigators have suggested that linoleic acid has anti-inflammatory potential by reducing IL-1 and IL-6 [34]. Furthermore, conjugated linoleic acid—an isomer of linoleic acid—reduced TNF-α concentration in RA patients and had an anti-inflammatory effect on active RA [35]. However, linoleic acid is also a precursor of the pro-inflammatory compounds AA and prostaglandin E2 (PGE2), which can aggravate inflammation [36]. Our results showed that linoleic acid was negatively correlated with disease activity, and the linoleic acid metabolic pathway was enhanced in the progression of RA, which may be related to the increase of downstream metabolites of linoleic acid. Although our differential metabolites were not enriched in AA metabolic pathway, PTGS1 mRNA expression level was significantly increased with the increase of disease activity, indicating that AA metabolic pathway was also involved in RA disease progression. Taken together, the study of lipid metabolism pathway changes has a potential role in RA progression, and maybe find a new therapeutic direction to improve the clinical reduction rate of RA.

Previous studies have shown that anti-rheumatic drug treatment of RA alters the transcriptome molecular profile to be more similar to that of HCs [37]. Inamo et al. identified 9 and 23 genes associated with clinical remission in rheumatoid arthritis CD4 and CD8 T cells, respectively [38]. Similarly, in our transcriptomic study, we found an association between gene expression levels and DAS28 scores. This suggests that changes in RA disease activity can be reflected at the transcriptional level.

This study was the first to analyze the relationship between nsSNV and RA disease activity by WES. In PPI analysis, NCF1 have the largest number of nodes interacting with lipid metabolism genes. NCF1 is a 47-kDa cytosolic subunit of the nicotinamide adenine dinucleotide phosphate (NADPH) oxidase complex, which is physically related to protein arginine deiminases (PADs), a key enzyme in RA pathogenesis [39]. The mutation rate of NCF1 rs201802880 in the DAS28M and DAS28H groups was higher than that in the DAS28L group, indicating that missense mutation of NCF1 rs201802880 may be related to both susceptibility to rheumatic diseases and disease activity [40]. Moreover, multiple nsSNVs of HLA-DRB1 and HLA-DRB5 gene locus have similar results to NCF1 RS201802880. Allele variants of HLA-DRB1 and HLA-DRB5 were associated with RA risk and were positively associated with elevated ESR [41]. However, we have not found reports on the relationship between nsSNV of HLA-DRB1 and HLA-DRB5 and RA disease activity. Therefore, our findings may be another new field for studying the occurrence and development of RA.

Among the constructed models, the multi-omics model performance of the DAS28L vs. HC groups showed the best performance in the training and external validation cohort and a better result than the single-omics model [15]. Most of the characteristic parameters involved in the model construction were metabolites, including three metabolites in the glycerophospholipid metabolic pathway, among which glycerophosphocholine was the most important feature to distinguish the two groups. This suggests plasma metabolites are a good marker to distinguish between healthy and RA. The other two groups of models (DAS28H vs. DAS28M groups, DAS28M vs. DAS28L groups) have better performance in the discovery cohort with AUC value above 0.76, but the unsatisfactory performance of the external validation cohort. The most likely cause was the characteristic parameters selected for model construction consist almost entirely of bacteria and fungi, and the species importance of metabolites ranks lower. There were fewer metabolites of difference between disease groups, indicating intestinal microbes more favorable to distinguish between disease groups. Additionally, our study demonstrated plasma metabolites and intestinal microbes play complementary roles in distinguishing populations with different disease activity levels. The combined application of multi-omics could better assist the clinical evaluation of the disease status of RA patients.

There are several limitations in our study. Firstly, the discovery cohort of the DAS28L group included few study populations, which may miss some potential information. Secondly, all RA patients in our study came from the inpatient system, and although we analyzed the vast majority of comorbidities, we could not completely exclude the potential impact of other comorbidities on this study. Thirdly, we found features in the transcriptome that correlate with RA disease activity, and it is not clear to us whether these features have the same results at the protein level, which needs to be confirmed by data in proteomics, especially in synovial tissue. Finally, in the WES analysis, variant loci for some genes were found at increased frequencies in the moderate and high disease activity groups, and although this gene was reported to be associated with susceptibility to RA, this variant loci still needs to be validated in a larger cohort. Meanwhile, compared to whole genome analysis, WES may lose part of the variation information, including non-coding regions and structural variants. In the future, we will use the existing evidence to find more specific information from different dimensions to consolidate our findings.

## Conclusion

In summary, compared with single or two omics analysis methods, we combined multiple omics analysis methods to explore more detail of the changes of plasma metabolites, intestinal bacteria, and fungi in the progression of RA, as well as their relationship with disease activity. The potential role of lipid metabolic pathway alterations in RA progression may provide a possible novel therapeutic direction for improving the clinical remission rate of RA. Furthermore, the model constructed based on multi-omics could assist in the clinical evaluation of the disease status of RA patients.

## Supplementary Information

**Additional file 1.**

**Additional file 2.**

**Additional file 3.**

**Additional file 4.**

**Additional file 5.**

**Additional file 6.**

**Additional file 7.**

**Additional file 8.**

**Additional file 9.**

## Data Availability

The datasets generated and/or analyzed during the current study are not publicly available but are available from the corresponding author on a reasonable request.

## Abbreviations

RA: Rheumatoid arthritis
HC: Healthy controls
DAS28: Disease Activity Score 28
ITS: Internally Transcribed Spacer
LC-MS: Liquid chromatograph mass spectrometer
MADAs: Metabolites associated with disease activity
RA-seq: RA sequencing
WES: Whole exome sequencing
DEGs: Differentially expressed genes
nsSNV: Non-synonymous single nucleotide variation
ROC: Receiver operating characteristic curves
PPI: Protein-protein interaction
PICRUSt2: Phylogenetic Investigation of Communities by Reconstruction of Unobserved States
LefSe: Linear discriminant analysis (LDA) effect size
PMBC: Peripheral mononuclear blood cell
LMRGs: Lipid metabolism-related genes
PGE: Prostaglandin E
LT: Leukotriene
HETE: Hydroxyeicosatetraenoic acid

## References

[1] Aletaha D, Neogi T, Silman AJ, Funovits J, Felson DT, Bingham CO (2010). 2010 rheumatoid arthritis classification criteria: an American College of Rheumatology/European League Against Rheumatism collaborative initiative. Ann Rheum Dis.

[2] Smolen JS, Aletaha D, Barton A, Burmester GR, Emery P, Firestein GS (2018). Rheumatoid arthritis. Nat Rev Dis Primers.

[3] Aletaha D, Smolen JS (2018). Diagnosis and management of rheumatoid arthritis: a review. JAMA.

[4] Conigliaro P, Triggianese P, De Martino E, Fonti GL, Chimenti MS, Sunzini F (2019). Challenges in the treatment of rheumatoid arthritis. Autoimmun Rev.

[5] Aletaha D, Smolen JS (2002). The rheumatoid arthritis patient in the clinic:comparing more than 1300 consecutive DMARD courses. Rheumatology (Oxford).

[6] Anderson J, Caplan L, Yazdany J, Robbins ML, Neogi T, Michaud K (2012). Rheumatoid arthritis disease activity measures: American College of Rheumatology recommendations for use in clinical practice. Arthritis Care Res (Hoboken).

[7] Fransen J, van Riel PL. The Disease Activity Score and the EULAR response criteria. Rheum Dis Clin North Am. 2009;35(4):745–57, vii-viii. 10.1016/j.rdc.2009.10.001 19962619

[8] Yu C, Jin S, Wang Y, Jiang N, Wu C, Wang Q (2019). Remission rate and predictors of remission in patients with rheumatoid arthritis under treat-to-target strategy in real-world studies: a systematic review and meta-analysis. Clin Rheumatol.

[9] Li C, Chen B, Fang Z, Leng YF, Wang DW, Chen FQ (2020). Metabolomics in the development and progression of rheumatoid arthritis: a systematic review. Joint Bone Spine.

[10] He Z, Liu Z, Gong L (2021). Biomarker identification and pathway analysis of rheumatoid arthritis based on metabolomics in combination with ingenuity pathway analysis. Proteomics.

[11] Holers VM, Demoruelle MK, Kuhn KA, Buckner JH, Robinson WH, Okamoto Y (2018). Rheumatoid arthritis and the mucosal origins hypothesis: protection turns to destruction. Nat Rev Rheumatol.

[12] Xu H, Zhao H, Fan D, Liu M, Cao J, Xia Y (2020). Interactions between gut microbiota and immunomodulatory cells in rheumatoid arthritis. Mediators Inflamm.

[13] Okada Y, Diogo D, Greenberg JD, Mouassess F, Achkar WA, Fulton RS (2014). Integration of sequence data from a Consanguineous family with genetic data from an outbred population identifies PLB1 as a candidate rheumatoid arthritis risk gene. PLoS ONE.

[14] Ying Li, Leung Elaine Lai-Han, Pan Hudan, Yao Xiaojun, Huang Qingchun, Wu Min (2017). Identification of potential genetic causal variants for rheumatoid arthritis by whole-exome sequencing. Oncotarget.

[15] Sasaki C, Hiraishi T, Oku T, Okuma K, Suzumura K, Hashimoto M (2019). Metabolomic approach to the exploration of biomarkers associated with disease activity in rheumatoid arthritis. PLoS ONE.

[16] Chen J, Wright K, Davis JM, Jeraldo P, Marietta EV, Murray J (2016). An expansion of rare lineage intestinal microbes characterizes rheumatoid arthritis. Genome Med.

[17] Terato K, Waritani T, Fukai R, Shionoya H, Itoh H, Katayama K (2018). Contribution of bacterial pathogens to evoking serological disease markers and aggravating disease activity in rheumatoid arthritis. PLoS ONE.

[18] Sanders SJ, Murtha MT, Gupta AR, Murdoch JD, Raubeson MJ, Willsey AJ (2012). De novo mutations revealed by whole-exome sequencing are strongly associated with autism. Nature.

[19] Zhang X, Zhang D, Jia H, Feng Q, Wang D, Liang D (2015). The oral and gut microbiomes are perturbed in rheumatoid arthritis and partly normalized after treatment. Nat Med.

[20] Yu D, Du J, Pu X, Zheng L, Chen S, Wang N, et al. The gut microbiome and metabolites are altered and interrelated in patients with rheumatoid arthritis. Front Cell Infect Microbiol. 2022;11:763507. 10.3389/fcimb.2021.763507 PMC8821809 35145919

[21] Jandhyala SM, Talukdar R, Subramanyam C, Vuyyuru H, Sasikala M, Nageshwar RD (2015). Role of the normal gut microbiota. World J Gastroenterol.

[22] Gomes AC, Hoffmann C, Mota JF (2018). The human gut microbiota: metabolism and perspective in obesity. Gut Microbes.

[23] Chiang HI, Li JR, Liu CC, Liu PY, Chen HH, Chen YM, et al. An association of gut microbiota with different phenotypes in Chinese patients with rheumatoid arthritis. J Clin Med. 2019;8(11):1770. 10.3390/jcm8111770 PMC6912313 31652955

[24] Jeong Y, Kim JW, You HJ, Park SJ, Lee J, Ju JH, et al. Gut microbial composition and function are altered in patients with early rheumatoid arthritis. J Clin Med. 2019;8(5):693. 10.3390/jcm8050693 PMC6572219 31100891

[25] Li Q, Wang C, Tang C, He Q, Li N, Li J (2014). Dysbiosis of gut fungal microbiota is associated with mucosal inflammation in Crohn’s disease. J Clin Gastroenterol.

[26] Bertolini M, Ranjan A, Thompson A, Diaz PI, Sobue T, Maas K (2019). Candida albicans induces mucosal bacterial dysbiosis that promotes invasive infection. PLoS Pathog.

[27] Allert S, Förster TM, Svensson CM, Richardson JP, Pawlik T, Hebecker B, et al. Candida albicans-induced epithelial damage mediates translocation through intestinal barriers. mBio. 2018;9(3):e00915–18. 10.1128/mBio.00915-18 PMC5989070 29871918

[28] Cristofori F, Dargenio VN, Dargenio C, Miniello VL, Barone M, Francavilla R (2021). Anti-inflammatory and immunomodulatory effects of probiotics in gut inflammation: a door to the body. Front Immunol.

[29] Amdekar S, Singh V, Kumar A, Sharma P, Singh R (2013). Lactobacillus casei and Lactobacillus acidophilus regulate inflammatory pathway and improve antioxidant status in collagen-induced arthritic rats. J Interferon Cytokine Res.

[30] Pan H, Guo R, Ju Y, Wang Q, Zhu J, Xie Y (2019). A single bacterium restores the microbiome dysbiosis to protect bones from destruction in a rat model of rheumatoid arthritis. Microbiome.

[31] Li Y, Zhang SX, Yin XF, Zhang MX, Qiao J, Xin XH (2021). The gut microbiota and its relevance to peripheral lymphocyte subpopulations and cytokines in patients with rheumatoid arthritis. J Immunol Res.

[32] Sun Y, Chen Q, Lin P, Xu R, He D, Ji W (2019). Characteristics of gut microbiota in patients with rheumatoid arthritis in Shanghai. China Front Cell Infect Microbiol.

[33] Su J, Li S, Chen J, Jian C, Hu J, Du H (2022). Glycerophospholipid metabolism is involved in rheumatoid arthritis pathogenesis by regulating the IL-6/JAK signaling pathway. Biochem Biophys Res Commun.

[34] Rodrigues HG, Vinolo MA, Magdalon J, Vitzel K, Nachbar RT, Pessoa AF (2012). Oral administration of oleic or linoleic acid accelerates the inflammatory phase of wound healing. J Invest Dermatol.

[35] Aryaeian N, Djalali M, Shahram F, Djazayery A, Eshragian MR (2014). Effect of conjugated linoleic acid, vitamin E, alone or combined on immunity and inflammatory parameters in adults with active rheumatoid arthritis: a randomized controlled trial. Int J Prev Med.

[36] Jandacek RJ. Linoleic Acid: A Nutritional Quandary. Healthcare (Basel). 2017;5(2):25. 10.3390/healthcare5020025 PMC5492028 28531128

[37] Tasaki S, Suzuki K, Kassai Y, Takeshita M, Murota A, Kondo Y (2018). Multi-omics monitoring of drug response in rheumatoid arthritis in pursuit of molecular remission. Nat Commun.

[38] Inamo J, Suzuki K, Takeshita M, Kondo Y, Okuzono Y, Koga K (2021). Molecular remission at T cell level in patients with rheumatoid arthritis. Sci Rep.

[39] Zhou Y, An LL, Chaerkady R, Mittereder N, Clarke L, Cohen TS (2018). Evidence for a direct link between PAD4-mediated citrullination and the oxidative burst in human neutrophils. Sci Rep.

[40] Yokoyama N, Kawasaki A, Matsushita T, Furukawa H, Kondo Y, Hirano F (2019). Association of NCF1 polymorphism with systemic lupus erythematosus and systemic sclerosis but not with ANCA-associated vasculitis in a Japanese population. Sci Rep.

[41] Klimenta B, Nefic H, Prodanovic N, Jadric R, Hukic F (2019). Association of biomarkers of inflammation and HLA-DRB1 gene locus with risk of developing rheumatoid arthritis in females. Rheumatol Int.

