# Supplementary material for: Multi-omics profiling reveals potential alterations in rheumatoid arthritis with different disease activity levels
Source: Arthritis Res Ther. 2023 May 3;25:74. doi: 10.1186/s13075-023-03049-z (PMC10155393; doi:10.1186/s13075-023-03049-z)
Supplement: Supplementary file 1 — Additional file 1. [file 13075_2023_3049_MOESM1_ESM.pdf]

Supplementary Table 1 . The baseline characteristics in the discovery cohort.

Parameters	HC (n = 50)	DAS28L (n=10)	DAS28M (n=45)	DAS28H (n=76)	P
Age (y)	53.9±10.8	61.2±10.9	54.7±13.0	60.4±12.3	0.006
Sex, F, No. (%)	36 (72.0)	8 (80.0)	33 (73.3)	56(73.7)	0.871
RF (U/mL)	——	119.2±121.4	298.8±312.6	335.2±286.1	0.014
CRP (mg/L)	——	13.0±24.3	27.5±34.6	52.6±36.1	<0.001
ESR (mm/h)	——	25.9±17.3	64.2±30.7	77.2±26.5	<0.001
DAS28 score	——	2.7±0.4	4.3±0.5	6.6±1.0	<0.001
Drug					
Methotrexate	——	3	7	13	0.549
Leflunomide	——	4	6	11	0.127
Prednisone	——	4	7	11	0.144
Comorbidities					
Hypertension	——	5	7	18	0.068
Diabetes	——	1	0	4	0.173
Osteoarthritis	——	1	13	10	0.084
Hyperlipemia	——	0	2	4	1.000
Osteoporosis	——	4	11	23	0.569

Except where indicated otherwise, values are mean±SD. RF, Rheumatoid Factor; CRP, C-reactive protein; ESR, Erythrocyte Sedimentation Rate; DAS28 score, 28 Joint disease activity score.

Supplementary Table 2. The baseline characteristics in the validation cohort.

Parameters	HC (n = 20)	DAS28L (n=21)	DAS28M(n=23)	DAS28H(n=29)	*P*
Age (y)	58. 1± 12.0	60.4± 10. 1	62.4± 11.3	59.3±9.5	0.615
Sex, F, No. (%)	12 (60.0)	15 (71.4)	18 (78.3)	19(65.5)	0.595
RF (U/mL)	— —	266.9±401.9	268.8±302.4	350. 1±296.9	0.589
CRP (mg/L)	— —	12.3± 17.0	49.2±43.6	71.0±52.7	<0.001
ESR (mm/h)	— —	44.3±31.0	69.6±33.8	77.6±29.8	0.002
DAS28 score	— —	2.5±0.6	4. 1±0.6	6.8±0.7	<0.001

Except where indicated otherwise, values are mean± SD. RF, Rheumatoid Factor; CRP, C-reactive protein; ESR, Erythrocyte Sedimentation Rate; DAS28 score, 28 Joint disease activity score.

Supplementary Table 3. Characteristic metabolites of RA at different disease activity levels.

Cationic model			Anion model					
Identifier	Metabolite	Compound ID(KEGG)	Identifier	Metabolite	Compound ID(KEGG)	Identifier	Metabolite	Compound ID(KEGG)
C1	Acetylcarnitine	C02571	A1	L-Threonine	C00188	A11	Pregnenolone sulfate	C18044
C2	Choline	C00114	A2	Linoleic acid	C01595	A12	4-Nonylphenol	C14550
C3	1-Palmitoylglycerol	NA	A3	Deoxycholic acid	C04483	A13	Cholesteryl sulfate	C18043
C4	Epsilon-Caprolactam	C06593	A4	Docosahexaenoic acid	NA	A14	Erucic acid	C08316
C5	MG(18:2(9Z,12Z)/0:0/0:0)[rac]	NA	A5	1,4-Dihydroxybenzene	C00530	A15	2E-Eicosenoic acid	NA
C6	1-Oleoyl-sn-glycero-3-phosphocholine	C03916	A6	L-Tryptophan	C00078	A16	Nervonic acid	C08323
C7	1-Stearoyl-2-hydroxy-sn-glycero-3-phosphocholine	C04230	A7	D-galacturonic acid	C00333	A17	Confertifoline	NA
C8	Glycerophosphocholine	C00670	A8	Indoxyl sulfate	NA	A18	Hypoxanthine	C00262
			A9	Bisindolylmaleimide I	C11238	A19	L-Leucine	C00123
			A10	P-Cresol	C01468			

Supplementary Table 4. Fifteen genes associated with DAS28(ESR).

**Parameter**	Gene	Rho	Pvalue	Relation
DAS28	BEST3	0.278932766	0.000680265	positive
DAS28	LTK	-0.275953219	0.000780454	negtive
DAS28	MCCD1	-0.271941082	0.00093681	negtive
DAS28	SMIM35	-0.259835298	0.001598707	negtive
DAS28	OSBP2	0.244074635	0.00309152	positive
DAS28	SCART1	-0.237657164	0.003997486	negtive
DAS28	PRSS22	-0.236852871	0.004126429	negtive
DAS28	PZP	-0.234460496	0.004532332	negtive
DAS28	ZACN	-0.220280811	0.007759494	negtive
DAS28	PF4V1	0.209341233	0.011502532	positive
DAS28	PIK3R2	-0.206201159	0.012835487	negtive
DAS28	AHSP	0. 192419998	0.020411243	positive
DAS28	SPATA21	-0. 185479592	0.025512242	negtive
DAS28	KRT1	0. 180723789	0.029606742	positive
DAS28	DNAJB13	-0. 169078541	0.042051012	negtive

DAS28 (ESR), Disease Activity Score 28 based on ESR.
