# Supplementary material for: Multi-omics profiling reveals potential alterations in rheumatoid arthritis with different disease activity levels
Source: Arthritis Res Ther. 2023 May 3;25:74. doi: 10.1186/s13075-023-03049-z (PMC10155393; doi:10.1186/s13075-023-03049-z)
Supplement: Supplementary file 2 — Additional file 2. [file 13075_2023_3049_MOESM2_ESM.docx]

**Multi-omics profiling reveals potential alterations in rheumatoid arthritis with different disease activity levels**

**Methods for intestinal microbiota analysis**

**Sample Collection**

The first faeces samples of all participants were collected after enrollment and immediately transported to the laboratory.Divide stool samples into EP tubes according to standard procedures, at least 1ml per tube.The separated samples were placed in liquid nitrogen for 1 minute and immediately cold stored at -80℃.

**DNA extraction and PCR amplification**

Total DNA was extracted from all samples using the E.Z.N.A.® soil DNA kit (Omega Bio-tek, Norcross, GA, U.S.) according to the manufacturer's protocol. All DNA samples were tested for quality, and DNA concentration and purity were determined by NanoDrop 2000 UV-vis spectrophotometer (Thermo Fisher Scientific, Wilmington, DE, USA). The bacterial 16S rRNA gene fragment (V3-V4) was amplified from DNA extracted with primers 338F (5-ACTCCTACGGGAGGCAGCAGCAG-3) and 806R (5-GGACTACHVGG

GTWTCTAAT-3), using primer ITS1F (5-CTTGGTCATTTAGAGGAAGTAA-3) and ITS2R (5-GCTGCGTTCTTCTTCTTCATCGATGC-3) amplification fungal internal transcribed spacer (ITS) gene. Amplification was performed with ABI GeneAmp 9700 instrument according to PCR amplification protocol. PCR reaction system was as follows : 4 μL of *TransStart* FastPfu buffer, 2 μL of 2.5 mM deoxynucleoside triphosphates (dNTPs), 0.8 μL of each primer (5 μM), 0.4 μL of *TransStart* FastPfu DNA Polymerase, 10 ng of template DNA, ddH2O supplemented to 20 μL.

**Illumina MiSeq sequencing**

The PCR product was extracted from 2% agarose gel and purified using the AxyPrep DNA Gel Extraction Kit (Axygen Biosciences, Union City, CA, USA) according to manufacturer’s instructions and quantified using Quantus™ Fluorometer (Promega, USA). The NEXTflexTM Rapid DNA-Seq Kit (Bioo Scientific, USA) was used for library construction: (1) joint link; (2) remove the self-connecting segment of the joint; (3) PCR amplification and library template enrichment; (4) Recovery of PCR products. Sequencing using Illumina's Miseq PE300 platform (Shanghai Majorbio Bio-Pharm Technology Co. Ltd).

**Processing of sequencing data**

The original sequencing sequence was preprocessed using FASTP software （https://github.com/OpenGene/fastp，version 0.20.0）：(1) the truncated reads shorter than 20 bp were discarded; (2) The paired reads are spliced into a sequence, and the minimum overlap length is greater than 10bp；(3) The maximum mismatch ratio of the splicing sequence was 0.2, and the nonconforming sequence was removed; (4) Samples were distinguished according to barcode and primers of the sequence, and sequence orientation was adjusted. Barcode mismatch was not allowed, and primers were allowed to mismatch twice. All sequences were aligned on Silva 16S rRNA database (version 138) by RDP classifier (http://rdp.cme.msu.edu/，version 2.2), and the alignment threshold was set to 70%. Finally, the species classification annotation information of each sequence was obtained.
