# Supplementary material for: Multi-omics profiling reveals potential alterations in rheumatoid arthritis with different disease activity levels
Source: Arthritis Res Ther. 2023 May 3;25:74. doi: 10.1186/s13075-023-03049-z (PMC10155393; doi:10.1186/s13075-023-03049-z)
Supplement: Supplementary file 3 — Additional file 3. [file 13075_2023_3049_MOESM3_ESM.docx]

**Multi-omics profiling reveals potential alterations in rheumatoid arthritis with different disease activity levels**

**Metabolic analysis method**

**Metabolite extraction**

According to the metabolite extraction protocol, we accurately extracted 100ul of plasma for metabolite extraction. The extracted metabolites were transferred into sample vials for LC-MS/MS analysis. During the analysis, one QC sample was inserted into every 10 samples to ensure the repeatability of the entire analysis process.

**UPLC-MS/MS analysis.**

The 10ul sample was separated on a BEH C18 column (100 mm × 2.1 mm i.d., 1.7 µm; Waters, Milford,USA) and then entered for mass spectrometry. The chromatographic separation was performed on the ExionLCTMAD system (AB Sciex, USA). Mobile phase A: water (containing 0.1% formic acid), mobile phase B: acetonitrile : isopropanol (1:1)(containing 0.1% formic acid), the gradient of mobile phase separation was changed according to the test protocol. All samples were kept under 4 ℃ conditions during analysis. The UPLC system was coupled with the quadrupole time-of-flight mass spectrometer for sample quality spectrum signal acquisition, and the positive and negative ion scanning mode was adopted, and the mass scanning range was 50-1000m/z.

**Data preprocessing and annotation**

After preprocessing the original LC-MS data, a data matrix containing (RT), mass-to-charge ratio (m/z) values, and peak intensity was generated. The data matrix uses the 80% rule to remove missing values, that is, to retain metabolites with more than 80% nonzero values in at least one set of samples. Then the minimum value is used to fill the vacancy value, and the sum normalization method is used to process the data matrix. Meanwhile, the metabolites of relative standard deviation (RSD) of QC>30% were deleted to obtain the final data matrix for subsequent analysis.
