# Supplementary figures and images for: Multi-omics profiling reveals potential alterations in rheumatoid arthritis with different disease activity levels
Source: Arthritis Res Ther. 2023 May 3;25:74. doi: 10.1186/s13075-023-03049-z (PMC10155393; doi:10.1186/s13075-023-03049-z)

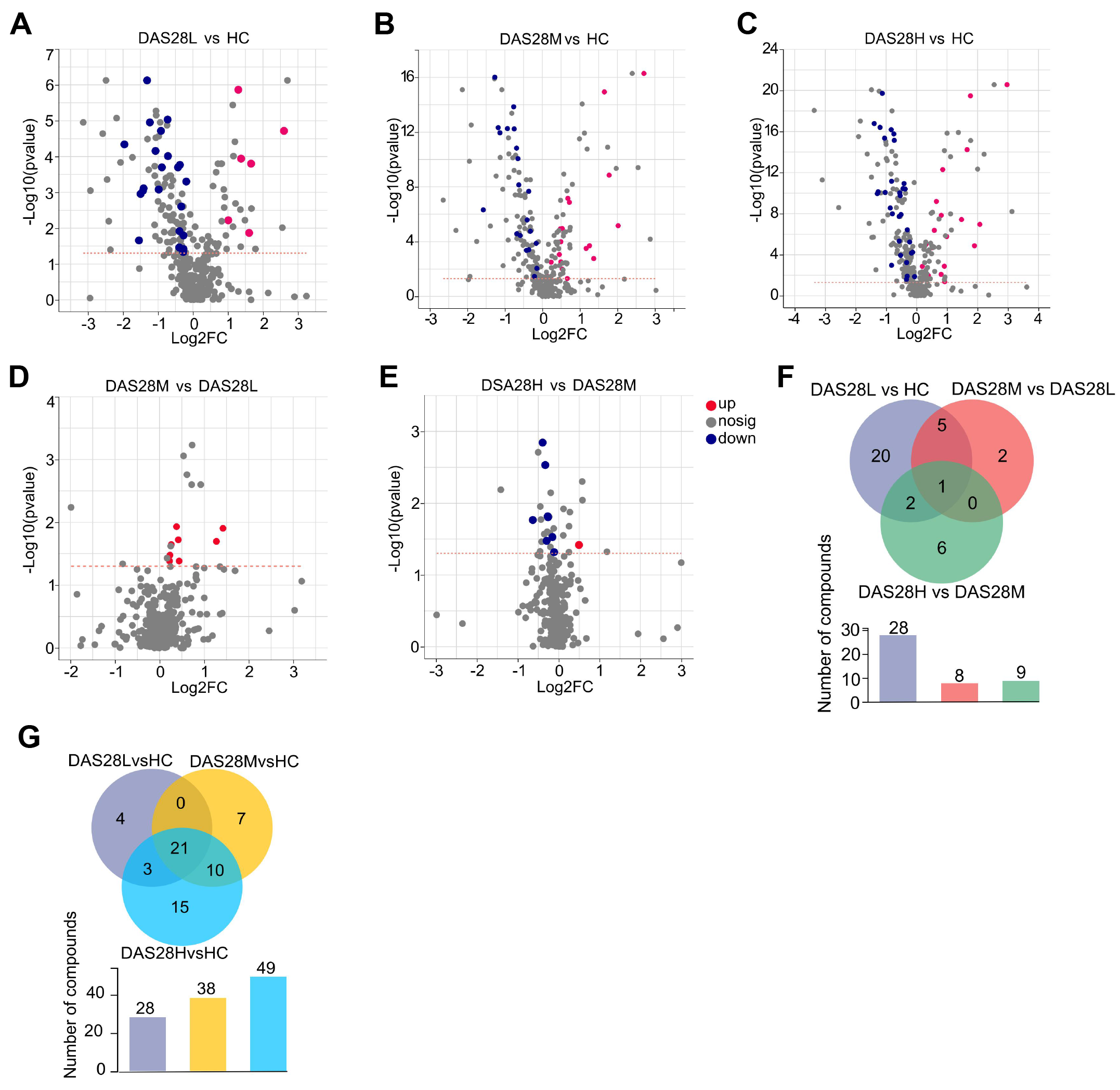

Supplement: Supplementary file 4 — Additional file 4. [file 13075_2023_3049_MOESM4_ESM.tif]

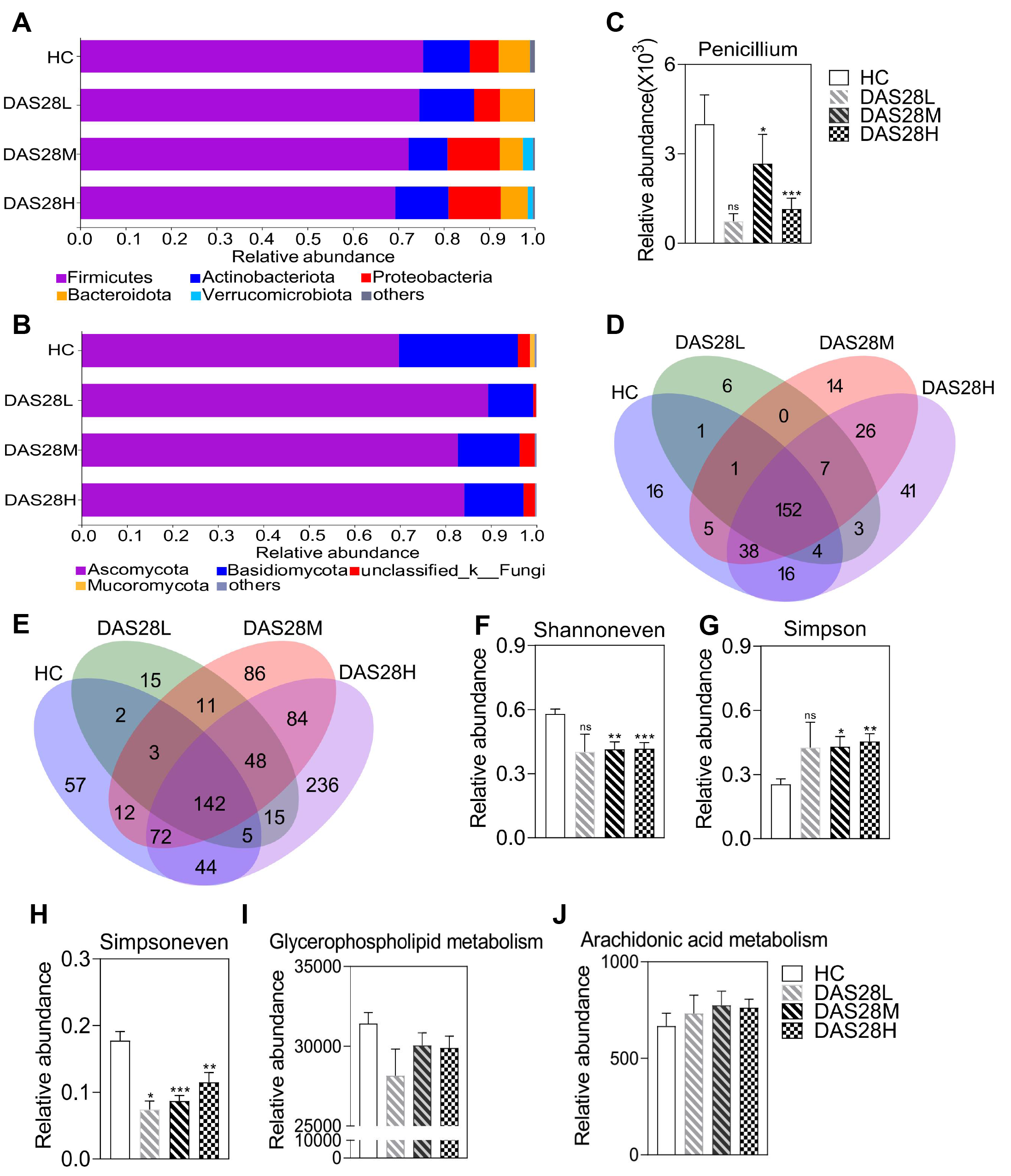

Supplement: Supplementary file 5 — Additional file 5. [file 13075_2023_3049_MOESM5_ESM.tif]

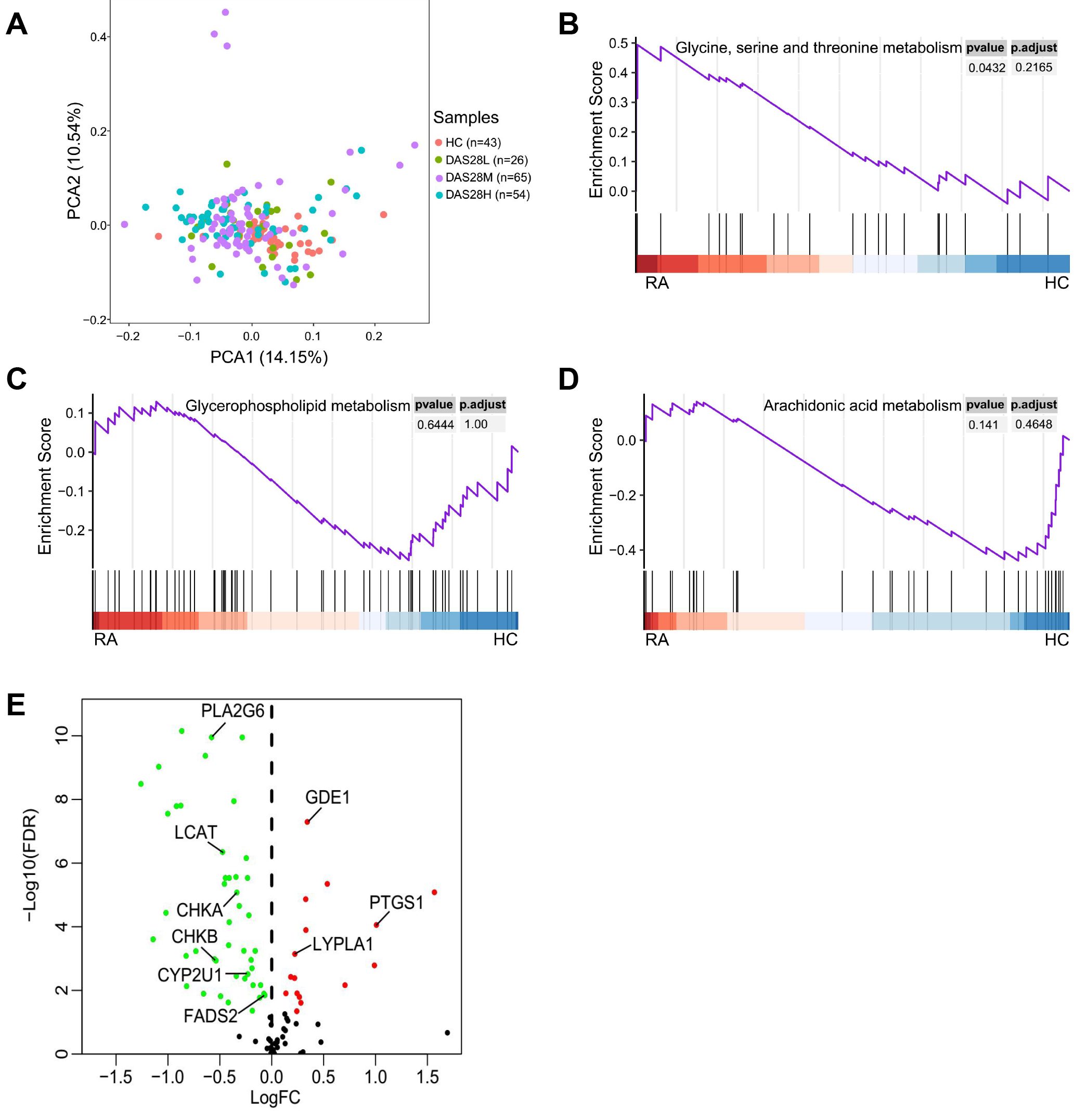

Supplement: Supplementary file 6 — Additional file 6. [file 13075_2023_3049_MOESM6_ESM.tif]

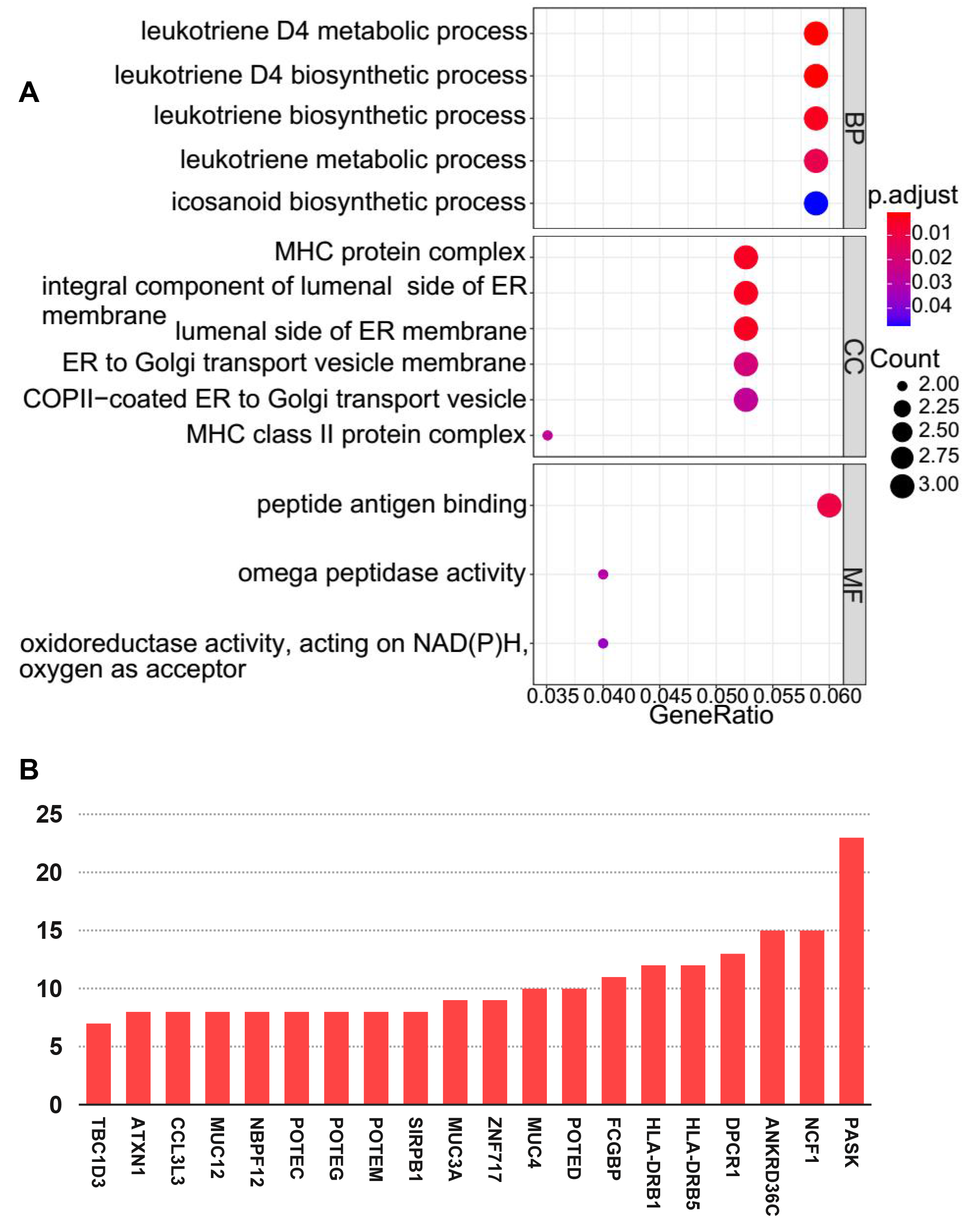

Supplement: Supplementary file 7 — Additional file 7. [file 13075_2023_3049_MOESM7_ESM.tif]

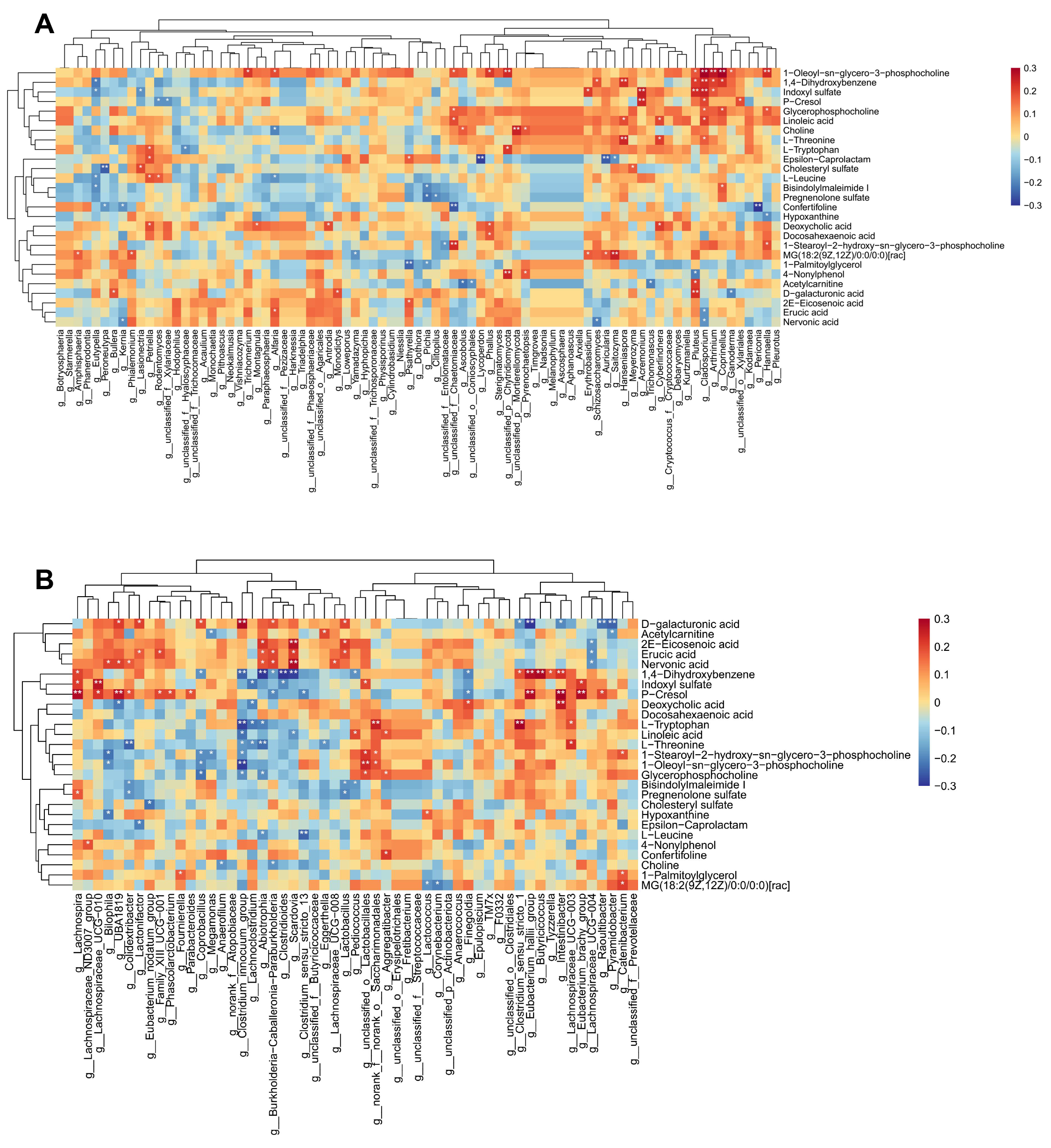

Supplement: Supplementary file 8 — Additional file 8. [file 13075_2023_3049_MOESM8_ESM.tif]

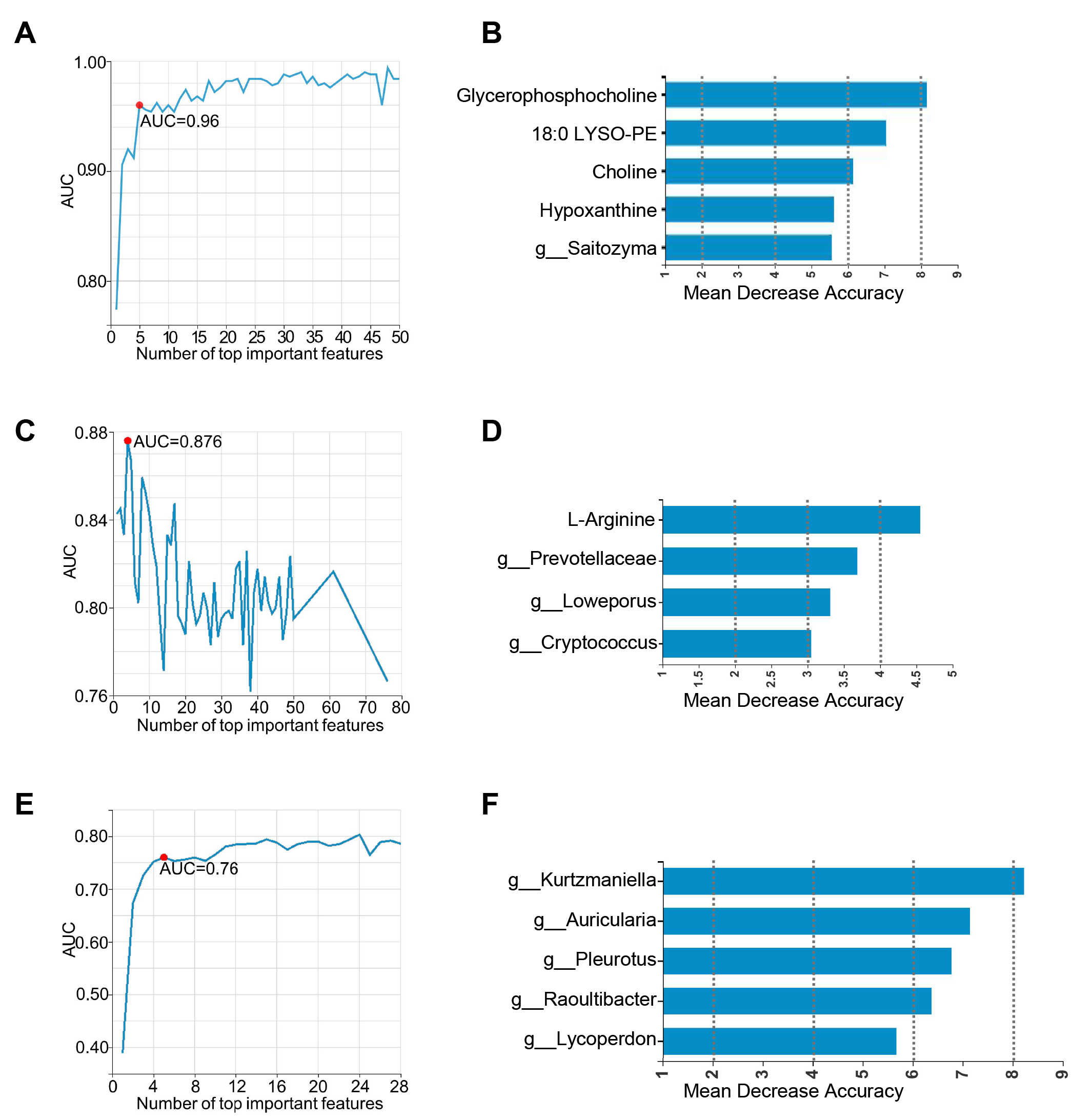

Supplement: Supplementary file 9 — Additional file 9. [file 13075_2023_3049_MOESM9_ESM.tif]
